# Germ layer-specific regulation of cell polarity and adhesion gives insight into the evolution of mesoderm

**DOI:** 10.7554/eLife.36740

**Published:** 2018-07-31

**Authors:** Miguel Salinas-Saavedra, Amber Q Rock, Mark Q Martindale

**Affiliations:** 1 The Whitney Laboratory for Marine Bioscience University of Florida Florida United States; 2 Department of Biology University of Florida Florida United States; Stowers Institute for Medical Research United States; University of Michigan United States

**Keywords:** Nematostella vectensis, mesoderm, epithelial-to-mesenchymal transition, cell polarity, cell adhesion, Other

## Abstract

In triploblastic animals, Par-proteins regulate cell-polarity and adherens junctions of both ectodermal and endodermal epithelia. But, in embryos of the diploblastic cnidarian *Nematostella vectensis*, Par-proteins are degraded in all cells in the bifunctional gastrodermal epithelium. Using immunohistochemistry, CRISPR/Cas9 mutagenesis, and mRNA overexpression, we describe the functional association between Par-proteins, ß-catenin, and *snail* transcription factor genes in *N. vectensis* embryos. We demonstrate that the aPKC/Par complex regulates the localization of ß-catenin in the ectoderm by stabilizing its role in cell-adhesion, and that endomesodermal epithelial cells are organized by a different cell-adhesion system than overlying ectoderm. We also show that ectopic expression of *snail* genes, which are expressed in mesodermal derivatives in bilaterians, is sufficient to downregulate Par-proteins and translocate ß-catenin from the junctions to the cytoplasm in ectodermal cells. These data provide molecular insight into the evolution of epithelial structure and distinct cell behaviors in metazoan embryos.

## eLife digest

Most animals – including birds, fish and mammals – have symmetrical left and right sides, and are known as bilaterians. During early life, the embryos of animals in this group develop three distinct layers of cells: the ectoderm (outer layer), the endoderm (inner layer), and the mesoderm (middle layer). These layers then go on to form the animal’s tissues and organs. The ectoderm produces external tissues, such as the skin and the nervous system; the endoderm forms internal tissues, like the gut; and the mesoderm creates all tissues in between, like muscles and blood.

Another, smaller group of animals, called cnidarians, do not have left and right sides. Instead, they have a ‘radial symmetry’, meaning they have multiple identical parts arranged in a circle. These animals – which include corals, jellyfish and sea anemones – only develop two distinct layers of cells, equivalent to the outer and inner layers of bilaterians. Cnidarians evolved before bilaterians, but their genetic material is equally complex. So why did these two groups evolve to have different layers of cells? And how exactly do animal embryos develop these distinct layers?

To address these questions, Salinas-Saavedra et al. studied embryos of the sea anemone *Nematostella vectensis*. Molecules called Par-proteins play an important role in controlling how cells behave and attach to one another (and therefore how they form layers). So, using a technique called immunohistochemistry to look inside cells, Salinas-Saavedra et al. examined these proteins in the two layers of cells in sea anemone embryos.

The experiments found that in the sea anemones, Par-proteins are arranged differently in cells that form the ‘skin’ compared to cells that form the ‘gut’. In other words, cells in the outer layer attach to one another in a different way than cells in the inner layer, where the Par-proteins are degraded by ‘mesodermal’ genes. The findings also show that these sea anemones have all they need to form a third middle layer of cells. Like bilaterians, they could potentially move cells in and out of sheets that line surfaces inside the body – but they do not naturally do this.

Understanding how animals form different layers of cells is important for scientists studying evolution and the development of embryos. However, it also has wider applications. For instance, some cells involved in developing the mesoderm are also involved in forming tumors. Future research in this area could help scientists learn more about how cancer-like cells form in animals.

## Introduction

Bilaterian animals comprise more than the 95% of the extant animals on earth and exhibit enormous body plan diversity (Martindale and Lee, 2013). One of the most important morphological features in bilaterian evolution is the emergence of the mesoderm, an embryological tissue that gives rise important cell types such as muscle, blood, cartilage, bone, and kidneys in the space between ectoderm and endoderm. The emergence of mesoderm clearly contributed to the explosion of biological diversity throughout evolution (Martindale and Lee, 2013; Martindale, 2005). Cnidarians (e.g. sea anemones, corals, hydroids, and ‘jellyfish’) are the sister group to bilaterians, and despite their surprisingly complex genomes (Putnam et al., 2007), do not possess a distinct mesodermal tissue layer. Instead, the gastrodermal lining to their gut cavity consists of a bifunctional endomesodermal epithelium with molecular characteristics of both bilaterian endodermal and myoepithelial mesodermal cells (Martindale and Lee, 2013; Technau and Scholz, 2003; Martindale et al., 2004; Jahnel et al., 2014). For example, *brachyury* and *snail*, among other genes, contribute to the specification of the endomesodermal fates in both bilaterian and cnidarian embryos (Technau and Scholz, 2003; Martindale et al., 2004; Magie et al., 2007; Yasuoka et al., 2016; Servetnick et al., 2017). Yet in bilaterians, mesodermal cells segregate from an embryonic endomesodermal precursor to form both endoderm and a third tissue layer (mesoderm) not present in the embryos of diploblastic cnidarians (Martindale et al., 2004; Rodaway and Patient, 2001; Davidson et al., 2002; Maduro and Rothman, 2002; Solnica-Krezel and Sepich, 2012). How mesodermal cells originally segregated from an ancestral endomesodermal epithelium during animal evolution is still unclear (Martindale and Lee, 2013; Martindale, 2005; Technau and Scholz, 2003), particularly because virtually all of the genes required for mesoderm formation are present in cnidarian genomes (Putnam et al., 2007; Baumgarten et al., 2015; Chapman et al., 2010; Shinzato et al., 2011). During the last decade, several studies have described molecular and cellular characteristics related to the segregation of mesoderm during bilaterian development (Solnica-Krezel and Sepich, 2012; Keller et al., 2003; Darras et al., 2011; Schäfer et al., 2014). Here, we investigate the cellular basis of morphogenesis during embryogenesis of the ‘diploblastic’ sea anemone, *Nematostella vectensis*.

In most bilaterian embryos described to date, after a series of synchronous and stereotyped cleavage divisions, maternal determinants induce the localization of nuclear ß-catenin to blastomeres derived from the vegetal pole (Martindale and Lee, 2013). Hence, gastrulation and the specification of endomesodermal fates is restricted to the vegetal pole. In these species, *brachyury* is expressed at the border of the blastopore and *snail* is expressed in the prospective mesodermal tissues (Technau and Scholz, 2003). The formation of mesoderm involves a variety of cellular processes including the downregulation of E-cadherin, loss of apicobasal cell polarity, and in some cases, the induction of epithelial-to-mesenchymal transition (EMT) (Solnica-Krezel and Sepich, 2012; Schäfer et al., 2014; Acloque et al., 2009; Lim and Thiery, 2012).

Embryos of the cnidarian starlet sea anemone *N. vectensis* develop without a stereotyped cleavage pattern but cell fates become organized along the embryonic animal-vegetal axis (Fritzenwanker et al., 2007; Salinas-Saavedra et al., 2015). During blastula formation, embryonic cells of *N. vectensis* form a single hollow epithelial layer. Epithelial cells of the animal pole, characterized by the nuclear localization of *Nv*ß-catenin prior to gastrulation (Wikramanayake et al., 2003; Lee et al., 2007), invaginate by apical constriction to form the endomesodermal epithelium (Magie et al., 2007; Tamulonis et al., 2011). The expression of *Nvbrachyury* around the presumptive border of the blastopore and *Nvsnail* genes in the presumptive endomesodermal gastrodermis of *N. vectensis* embryos occurs even before the morphological process of gastrulation begins (Scholz and Technau, 2003; Röttinger et al., 2012).

**Figure 1.**
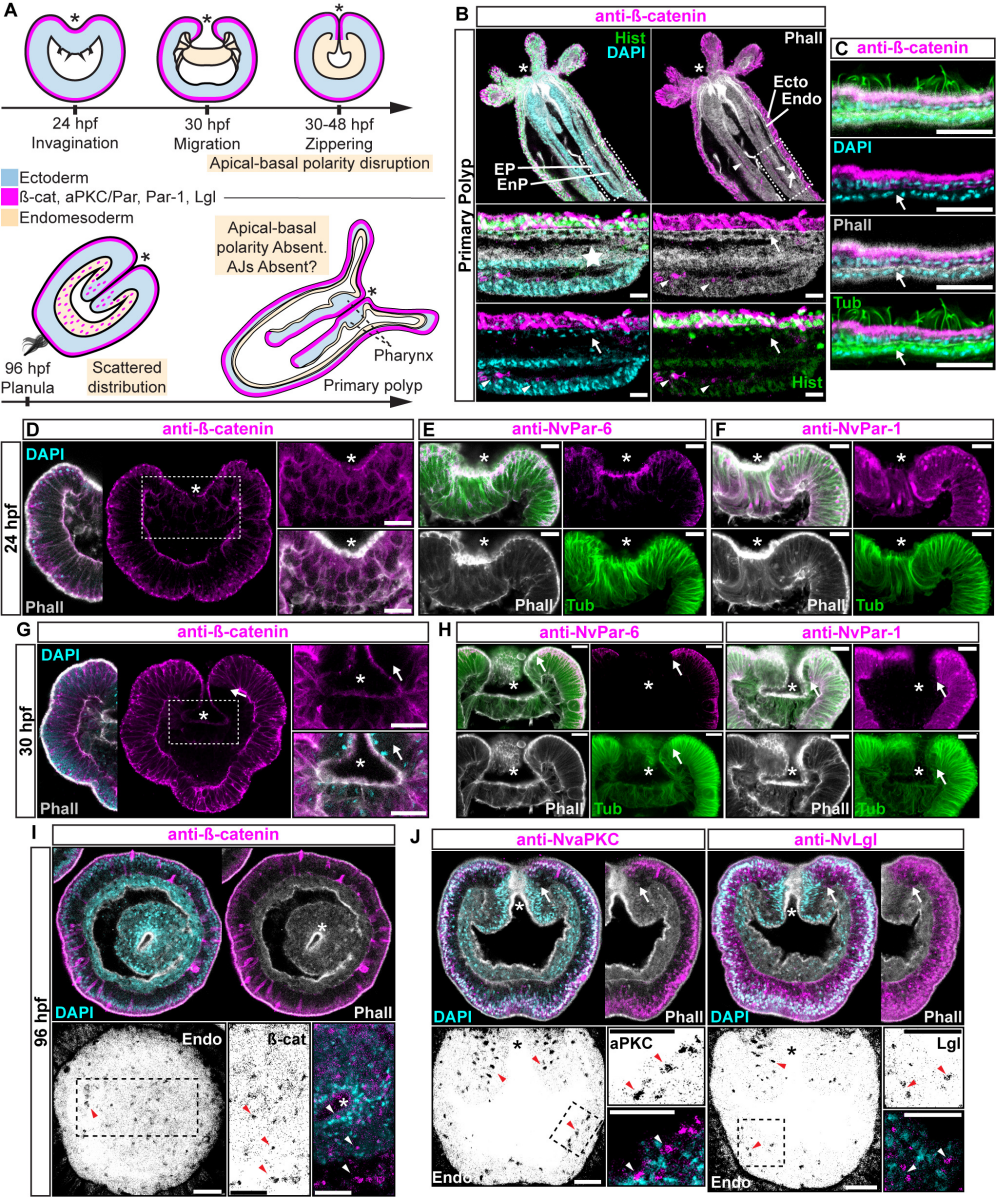
Components of the Par system and ß-catenin are downregulated from the *N. vectensis* endomesoderm during gastrulation. (**A–F**) Confocal images of immunofluorescent staining (IFS) of lateral views of gastrulation embryos (animal pole up). The * marks the site of gastrulation in all cases. Samples are counterstained with Phalloidin (Phall) staining (white) to show cell boundaries, DAPI to visualize cell nuclei (blue), and Tubulin antibody (Tub) staining is shown as counterstain (green). All images are a single optical section from a z-stack confocal series. All scale bars, 20 µm. (**A**) Summary diagram depicting the localization of ß-catenin and Par proteins at the observed stages. Pale boxes denote changes observed in the endomesoderm. (**B**) IFS for ß-catenin (magenta) in primary polyps. High magnification images from boxed region (endomesoderm, Endo) are shown on the bottom. Arrows indicate the absence of ß-catenin expression in the endomesoderm. Arrowheads indicate the ß-catenin expression in the ectodermal pharynx (EP). Star indicates the endomesodermal pharynx (EnP). Histone antibody (Hist) staining is shown as counterstain to show the penetrability in the fixed tissue. See also Figure 1—figure supplement 1. (**C**) IFS for ß-catenin (magenta) in the ecto and endomesoderm (arrow) of primary polyps. (**D**) IFS for ß-catenin (magenta) at 24 hpf shows localization to the apical domain where adherens junctions reside in all cells of the blastula. High magnification images from boxed region (prospective endomesoderm) are shown on the right. (**E**) IFS for *Nv*Par-6 (magenta) at 24 hpf showing the same sub-cellular localization as ß-catenin (**A**). High magnification images from boxed region in (**A**) (prospective endomesoderm) are shown on the right. Merged image shown on upper left. (**F**) IFS for *Nv*Par-1 at 24 hpf shows a complementary basolateral expression. High magnification images from boxed region (prospective endomesoderm) are shown on the right. (**G**) IFS for ß-catenin at 30 hpf shows the loss of expression of ß-catenin (magenta) in invaginating endomesoderm (box). The arrow (**D–F**) marks the boundary between ectoderm and invaginating endomesoderm. High magnification images from boxed region (prospective endomesoderm) are shown on the right. (**H**) IFS for *Nv*Par-6 and *Nv*Par-1(magenta) at 30 hpf show that all Par proteins are down regulated at the site of gastrulation. IFS for *Nv*Par-6 shows an even earlier down regulation than ß-catenin (**D**). High magnification images from boxed region (prospective endomesoderm) are shown on the right. Merged image shown on upper left. (**I**) Oral view of IFS for ß-catenin (magenta) at 96 hpf showing apical localization in overlying ectoderm, but absence in endomesodermal tissues. The two bottom panels show high magnifications of the endomesoderm region (image inverted). Arrowheads indicate the localization of ß-catenin expression (black) in some scattered endomesodermal cells. (**J**) Lateral view of IFS for *Nv*aPKC and *Nv*Lgl (magenta) at 96 hpf showing loss of expression in invaginating epithelial cells. The four bottom panels show high magnifications of the endomesoderm region (image inverted). Arrowheads indicate the localization of *Nv*aPKC and *Nv*Lgl proteins (black) in some scattered endomesodermal cells.

Interestingly, the components of the intracellular polarity Par system (*Nv*aPKC, *Nv*Par-6, *Nv*Par-3, *Nv*Par-1, and *Nv*Lgl), which show a highly polarized bilaterian-like subcellular distribution throughout all epithelial cells at the blastula stage in *N. vectensis* (Salinas-Saavedra et al., 2015), are specifically degraded and down-regulated from the endomesoderm during the gastrulation process (Figure 1A). We have previously suggested that the expression of bilaterian ‘mesodermal genes’ (e.g. *Nvsnail)* might induce the loss of apicobasal cell-polarity indicated by the absence of the components of the Par system in the endomesoderm of *N. vectensis* embryos (Salinas-Saavedra et al., 2015). Recent studies in *N. vectensis* and bilaterians have provided information that supports this hypothesis. For example, it has been shown that *snail* is necessary and sufficient to downregulate Par3 in *Drosophila* mesoderm, inducing the disassembly of junctional complexes in these tissues (Weng and Wieschaus, 2016, 2017). In addition, we have shown that *Nvbrachyury* regulates epithelial apicobasal polarity of *N. vectensis* embryos, suggesting some aspects of epithelial cell polarity are highly conserved (Servetnick et al., 2017). Together, this evidence suggests a plausible cellular and molecular mechanism for the segregation of a distinct cell layer in bilaterian evolution from an ancestral bifunctional endomesodermal tissue. Thus, in this study, we describe the functional association between the components of the Par system, apical junctions, epithelial integrity, and the nuclearization of *Nv*ß-catenin in a cnidarian embryo. In addition, we demonstrate that the endomesoderm in *N. vectensis* is organized by different junctional complexes that confer different functional properties to this tissue than the overlying ectoderm. And finally, we investigate the putative interactions between the components of the Par system, the canonical Wnt signaling pathway, and *snail* gene expression, giving insights on the evolution of the mesoderm and EMT.

**Figure 1—figure supplement 1.**
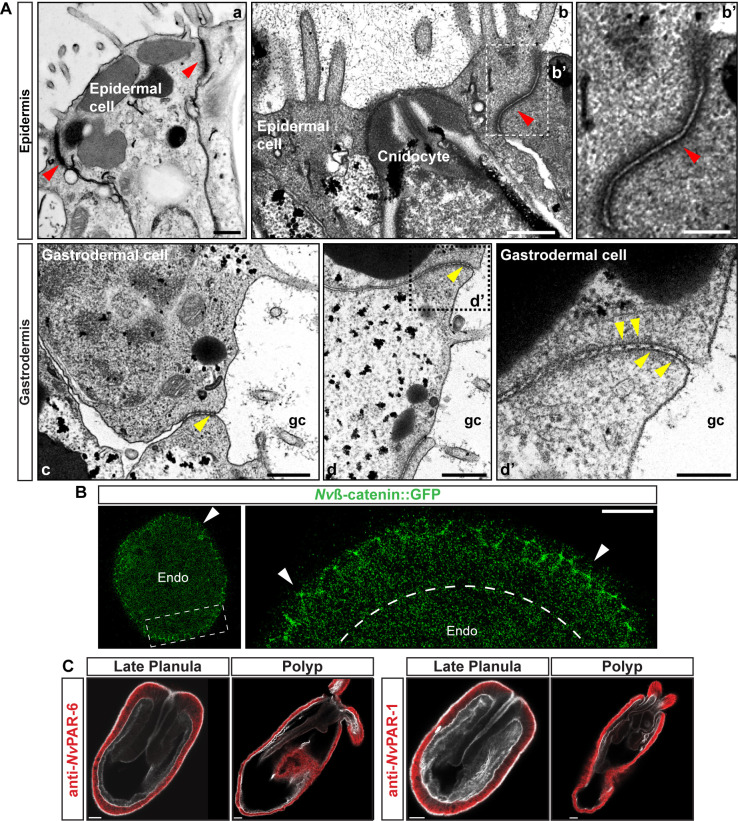
Epidermal and gastrodermal cells are joined by different set of junctional complexes. (**A**) TEM micrographs of *N. vectensis* primary polyps. Epidermal cells (ectodermally derived) are joined most likely by AJs (Red arrowheads). Gastrodermal cells (endomesodermally derived) are interconnected by fewer and shorter contacts, most likely by septate junctions (yellow arrowheads). a and b: two different types of epidermal cells. Note the ectodermally-derived cnidocyte in b. c and d: two different types of gastrodermal cells. b’ and d’ are high magnification images from boxed region in b and d, respectively. gc: gastric cavity. Scale bars in a, b, c, and d: 500 nm. Scale bars in b’ and d’: 200 nm. (**B**) *in vivo* localization of *Nv*ß-catenin::GFP after 36 hpf in *N. vectensis* embryos. Arrowheads indicate the cortical localization of *Nv*ß-catenin::GFP (AJs) in the ectoderm that was not observed in the endomesoderm. High magnification image from boxed region is shown on the right. Scale bars: 20 µm. (**C**) Immunofluorescent staining for *Nv*Par-6 and *Nv*Par-1(red) at late planula and polyp stages show that both Par proteins are absented from the endomesoderm. Phalloidin is shown in gray. Histone and tubulin antibody staining are shown in Figure 1B and C as counterstain to show the penetrability in the fixed tissue. Scale bars: 20 µm.

## Results

### Ectodermal and endomesodermal epithelia are organized by different cell-cell adhesion complexes

Components of the Par system are not present in the cells of endomesodermal epithelium of *N. vectensis* during gastrulation, even though the very same cells express these proteins during the blastula stage (Salinas-Saavedra et al., 2015) (Figure 1). This absence is consistent with the absence of apical Adherens Junctions (AJs) in the endomesoderm of *N. vectensis* (Figure 1—figure supplement 1) and other cnidarians (Magie et al., 2007; Chapman et al., 2010; Ganot et al., 2015). At polyp stages, neither ß-catenin (an AJ-associated protein) (Figure 1B and C) nor the Par proteins (Figure 1—figure supplement 1C) are detectable in endomesodermal cells of either the gastrodermis or the pharynx. When *N. vectensis* embryos are stained with antibodies to ß-catenin (Figure 1) or if *Nv*ß-catenin::GFP mRNA is expressed in uncleaved zygotes (Figure 1—figure supplement 1B), clear localization of ß-catenin can be seen in the cortex of ectodermally derived epithelial cells (Figures 1B, C, D, G and I), but not in endomesodermal cells (Figure 1B and C). In pharyngeal cells that are located between the epidermis and gastrodermis, *Nv*ß-catenin (Figure 1B and D), *Nv*Par-6 (Figure 1E), and *Nv*Par-1 (Figure 1F) expression begins to disappear, and is localized only in the most apical regions, indicating that AJs are being disassembled/degraded during the gastrulation process (Figure 1G and H). During later planula stages, ß-catenin and the components of the Par system display scattered patterns in the cytoplasm of a small subset of endomesodermal cells (Figure 1I and J). Even though we do not know the identity of these cells, this expression temporally coincides with the transient activation of Wnt signaling emanating from the oral pole (Kusserow et al., 2005; Marlow et al., 2013) at those developmental stages. In bilaterians (Acloque et al., 2009; Lim and Thiery, 2012) and *N. vectensis* (Kusserow et al., 2005; Marlow et al., 2013), the later activation of Wnt signaling is also associated with neurogenesis, and may cause the observed changes in protein localization.

**Figure 2.**
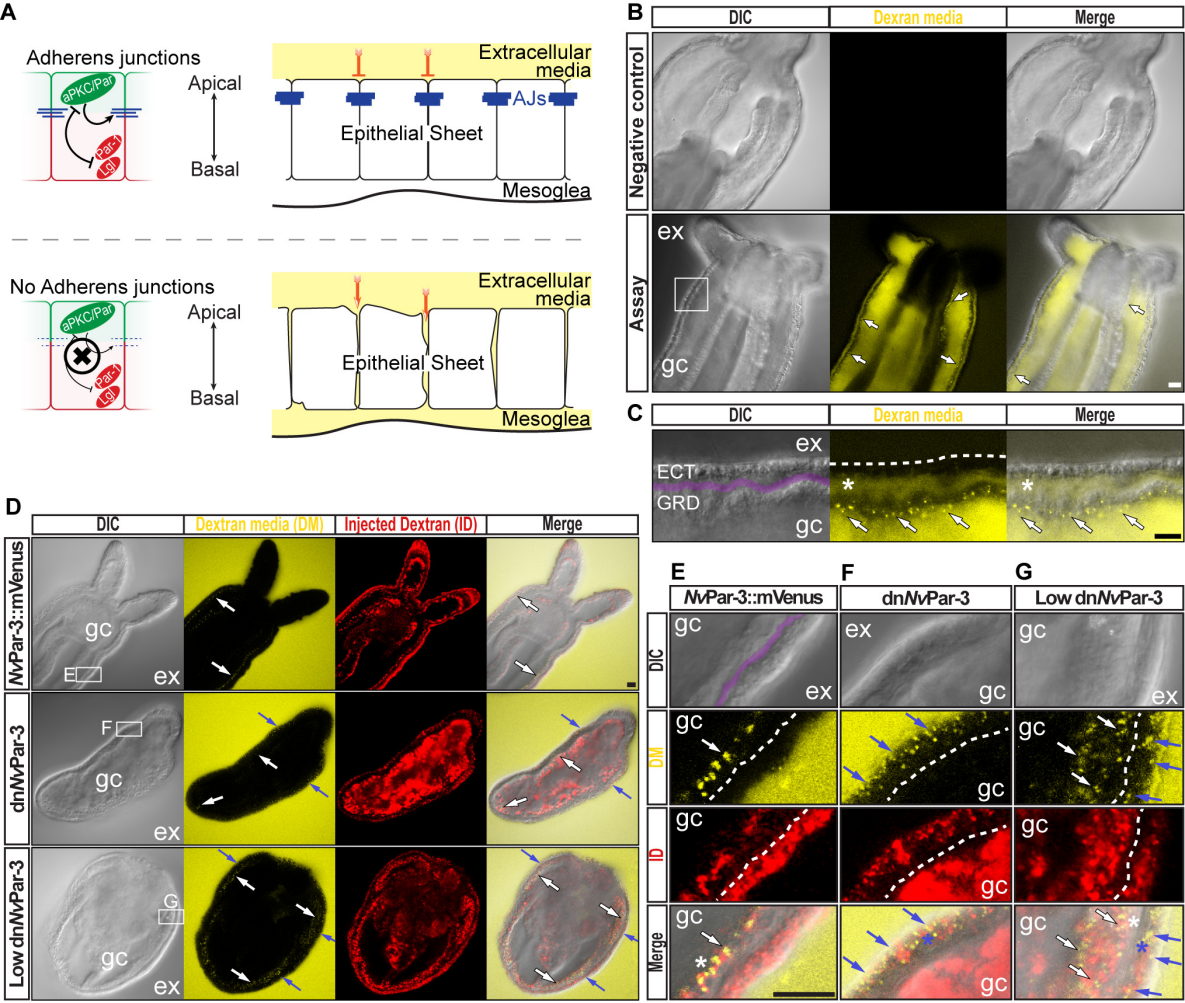
The aPKC/Par complex maintains Adherens Junctions (AJs) of ectodermal epithelial cells. Arrows indicate the direction of the flow: from gastric cavity (gc) to mesoglea (white) and from external media (ex) to ectoderm (blue). Dashed lines indicate the base of the epidermis. All images are single optical section from the z-stack confocal series. Scale bars, 20 µm. (**A**) Diagram depicting the hypothesis that when the aPKC/Par complex is functional (top row), AJs are present (blue stripes) and a paracellular epithelial barrier is formed. When aPKC/Par complex is not functional (bottom row), AJs are disrupted, the epithelial barrier is perturbed, and the extracellular solution moves paracellularly into the mesoglea. (**B**) Penetration assay of wild type (uninjected) primary polyps at low magnification showing the movement of 10,000 MW fluorescent dextran. Top row, no dextran. Bottom row, dextran (yellow) in the gc moves in to the mesoglea through paracellular spaces between gastrodermal cells (arrows). (**C**) High magnification images from box shown in (**B**). *: mesoglea (purple band) that separates the ectoderm (ECT, dashed line) from gastrodermis (GDR). Note the dye moving between cells from the gc media (arrows). (**D**) Low magnification images comparing polyps expressing *Nv*Par-3::mVenus and a dominant negative version of *Nv*Par-3 (dn*Nv*Par-3::mVenus) expressing-embryos. Dextran media (DM; extracellular) is pseudo-colored yellow. Dextran (red) was co-injected with mRNAs to label the cells and differentiate intracellular regions. mVenus channel was omitted for better visualization (shown in Figure 2—figure supplement 1). Lower concentrations of dn*Nv*Par-3 were injected to preserve endomesodermal tissues. Note that the dextran media was found between the cells labeled in red. See also Figure 3—figure supplement 1 for dn*Nv*Par-3 description. (**E**) High magnification images from (**E**) boxed region in (**D**). Purple band depicts Mesoglea. (**F**) High magnification images from (**F**) boxed region in (**D**). (**G**) High magnification images from (**G**) boxed region in (**D**). *: Paracellular spaces of both, the epidermis (blue) and gastrodermis (white).

Regardless of this scattered expression, it is clear that cells that undergo gastrulation in *N. vectensis* lose their polarized ectodermal cell-cell adhesion complex and components of the Par system, including ß-catenin, are downregulated from endomesodermal tissues (Figure 1). In bilaterians, the proper formation of an epithelial paracellular barrier (essential for tissue homeostasis) depends on the establishment of adhesive complexes between adjacent cells (Higashi et al., 2016; Jonusaite et al., 2016), which are regulated by the aPKC/Par complex (Ohno et al., 2015). To test if this absence of protein expression is correlated to differential cell-cell adhesion in the endomesodermal epithelium of *N. vectensis*, we assessed their role in regulating paracellular movements between ectodermal and endomesodermal epithelia by using a fluorescent tracer dye penetration assay (Figure 2A) (Higashi et al., 2016). For the purposes of these experiments, in order to avoid unwanted results related to tissue specification, cell proliferation, and cell movements, we used newly hatched juvenile polyps where the gastrodermis (endomesodermally derived) is fully differentiated.

**Figure 2—figure supplement 1.**
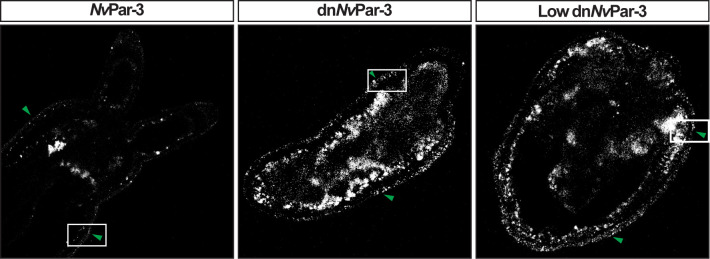
mVenus protein expression related to Figure 2. mVenus channel for *Nv*Par-3::mVenus (green arrow) and dn*Nv*Par-3::mVenus (green arrow) expressing-embryos shown in Figure 2. Laser intensity and gain were increased to allow visualization; however, signal noise from other channels and animal autofluorescence were also increased.

*N. vectensis* polyps were exposed to media containing 10,000 MW fluorescent dextran (Molecular Probes, Inc.). When juvenile polyps are incubated in dextran for 5–10 min (Figure 2B), fluorescent dextran solution moves into the gastric cavity and then spreads into the mesoglea through the gastrodermal epithelium (Figure 2C) but does not enter the mesoglea through the outer ectodermally-derived epidermis (Figure 2C and D). These results suggest that cell-cell adhesion is differentially regulated between the epidermis and gastrodermis and the absence/disruption of AJs may compromise Septate Junctions (SJs) in the gastrodermis. Similar results were obtained in *N. vectensis* polyps when we overexpressed *Nv*Par-3::mVenus by injection of mRNA into uncleaved eggs which is normally expressed in ectodermal but not endodermal epithelial tissue (Figure 2D and E). However, in polyps expressing a dominant negative version of *Nv*Par-3::mVenus (dn*Nv*Par-3; microinjected into uncleaved eggs) dye penetrated between epithelial cells in both the gastrodermis and the outer epidermis (Figures 2D, F and G), demonstrating an ancestral role of the aPKC/Par complex in the maintenance of cell-cell adhesion and the paracellular boundary (SJs) of epithelial cells during animal development.

### The *Nv*aPKC/Par complex regulates the formation and maintenance of cell-cell junctions

**Figure 3.**
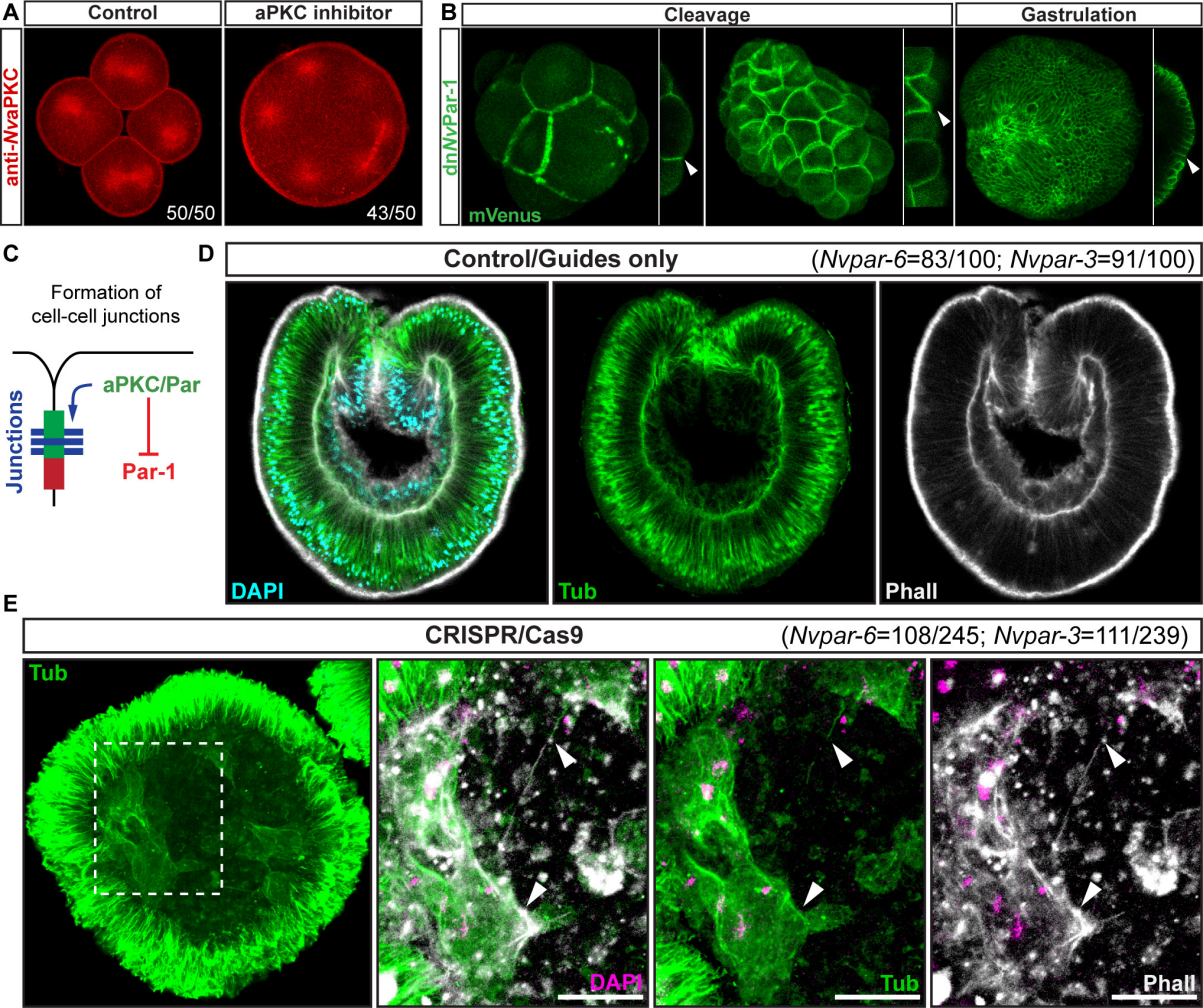
Ectodermal *Nv*aPKC/Par complex polarity regulates the epithelial integrity of both ecto- and endomesoderm. (**A**) IFS for *Nv*aPKC at 4 hpf showing that the aPKC inhibitor (Sigma P1614) blocks cytokinesis but not cell cycle. (**B**) *in vivo* expression of dn*Nv*Par-1 shows precocious localization to zones of cell contact during cleavage stages, well before wild-type *Nv*Par genes do. See also Figure 3—figure supplement 1A and Figure 3—figure supplement 2. (**C**) Diagram depicting the suggested the working hypothesis. (**D**) CRISPR/Cas9 knock-out for *Nv*Par-6 and *Nv*Par-3 at 48 hpf. Controls show no effect on gastrulation. Tubulin (Tub), Phalloidin (Phall), and DAPI are used as counterstains. (**E**) CRISPR/Cas9 mutants: tubulin stained low magnification of CRISPR phenotype. High magnification images from boxed region shows mesenchymal-like cells. Arrowheads indicate filopodia-like structures. Number of cases observed for each gene are shown. See also Figure 3—figure supplement 1, Figure 3—figure supplements 3–6, and Figure 3—video 1. Morphology is shown by DAPI, Tub, and Phall IFS. Except for 3B and 3D, all images are single optical sections from the z-stack confocal series. (**B**) and (**D**) are 3D reconstructions from a z-stack confocal series. All scale bars, 20 µm.

Our results suggest that the absence of Par proteins in the endomesoderm is associated with changes in cell-cell adhesion complexes. Pharmacological treatment of *N. vectensis* embryos with an aPKC activity inhibitor blocks cytokinesis but not mitosis in cleaving embryos (Figure 3A). In addition, a dominant negative version of *Nv*Par-1 (dn*Nv*Par-1), that lacks its kinase domain, localizes only to the cortex of cell–cell contacts (Figure 3B). Since *Nv*Par-1 is phosphorylated by *Nv*aPKC (Figure 3—figure supplement 2), we predict that, as in other systems, dn*Nv*Par-1 could be phosphorylated by aPKC but would not phosphorylate the aPKC/Par complex (Vaccari et al., 2005; Böhm et al., 1997). Thus, dn*Nv*Par-1 can localize to the cell cortex where aPKC may be inactive. These results together suggest that the formation of cell–cell contacts is regulated by the activity of the aPKC/Par complex in *N. vectensis* embryos (Figure 3C).

**Figure 3—figure supplement 1.**
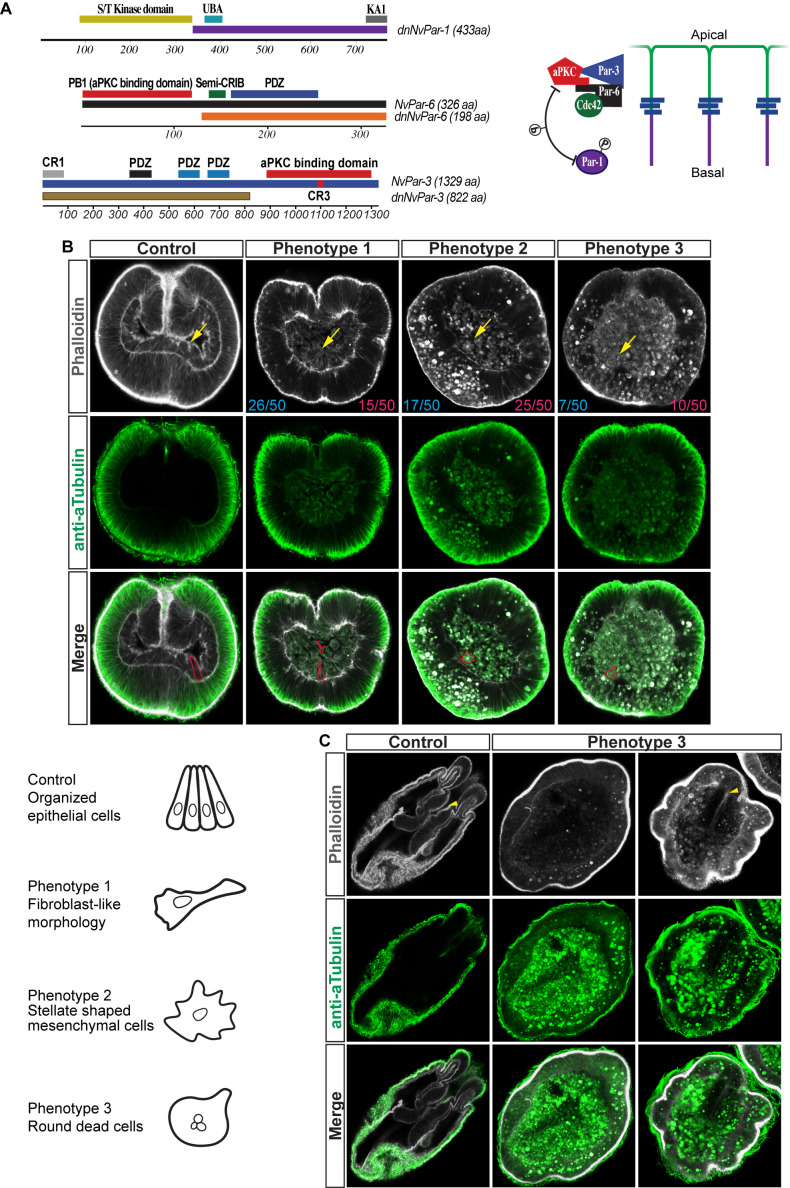
Disruption of the aPKC/Par complex in *N. vectensis* embryos. (**A**) Diagram depicting the modifications made to *Nv*Par-1, *Nv*Par-6, and *Nv*Par-3 sequence to generate the dominant negative version of each protein (dn*Nv*Par-1, dn*Nv*Par-6, and dn*Nv*Par-3, respectively), which lack the putative interaction domain with *Nv*aPKC. In the right, a diagram depicts the localization of *Nv*Par-1, *Nv*aPKC, *Nv*Par-6, and *Nv*Par-3 proteins in epithelial cells. The putative interaction with *Nv*aPKC, restricts the localization of *Nv*Par-6 and *Nv*Par-3 strictly to the apical cortex of the cell, and *Nv*Par-1 to the lateral cortex of the cell. See also Figure 3—figure supplements 5 and 6. (**B**) Immunofluorescent staining for Tubulin and Phalloidin at gastrula stage of embryos expressing dn*Nv*Par-6, and dn*Nv*Par-3. The overexpression of either dn*Nv*Par-6::mVenus or dn*Nv*Par-3::mVenus induced phenotypes where the endomesodermal cells (yellow arrows) are disorganized during gastrulation. We observed a disorganized endoderm formed by (1) cells with fibroblast-like morphologies, (2) stellate shaped mesenchymal-like cells, or (3) a mass of round dead cells. Cell morphotypes are outlined in red and their schematic representation is presented below them. The penetrance of the obtained phenotypes when either dn*Nv*Par-6::mVenus (blue) or dn*Nv*Par-3::mVenus (magenta) are overexpressed is indicated for each case. (**C**) Immunofluorescent staining for Tubulin and Phalloidin at polyp stage of embryos expressing dn*Nv*Par-6, and dn*Nv*Par-3. We observed ‘Endoderm-less’ polyps from phenotype 3: absence of an organized endoderm, tentacles or complete mesenteries in injected animals that survived to this stage (2 weeks post fertilization). A recognizable ectodermal pharynx (yellow arrowheads) was detected in some of these polyps.

**Figure 3—figure supplement 2.**
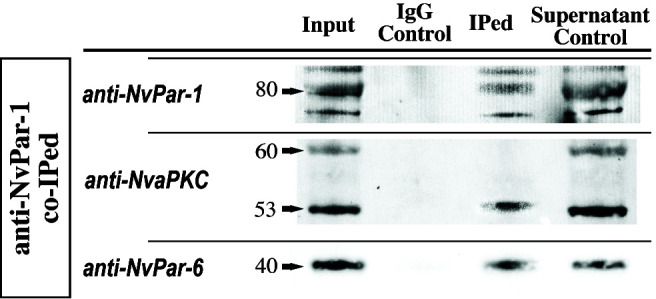
Co-immunoprecipitation of NvPar proteins using a NvPar-1 specific antibody. Co-IP experiments show that *Nv*Par-1 interacts with *Nv*aPKC and *Nv*Par-6 during *N. vectensis* development. Three bands labeled with NvPar antibody were observed around 80 KD, suggesting different phosphorylation states of this protein that may be product −1 of NvaPKC activity. Negative (IgG line) and Positive controls (Input and Supernatant) are also shown. Numbers and arrowheads in the first column indicate the molecular weight in KD.

**Figure 3—figure supplement 3.**
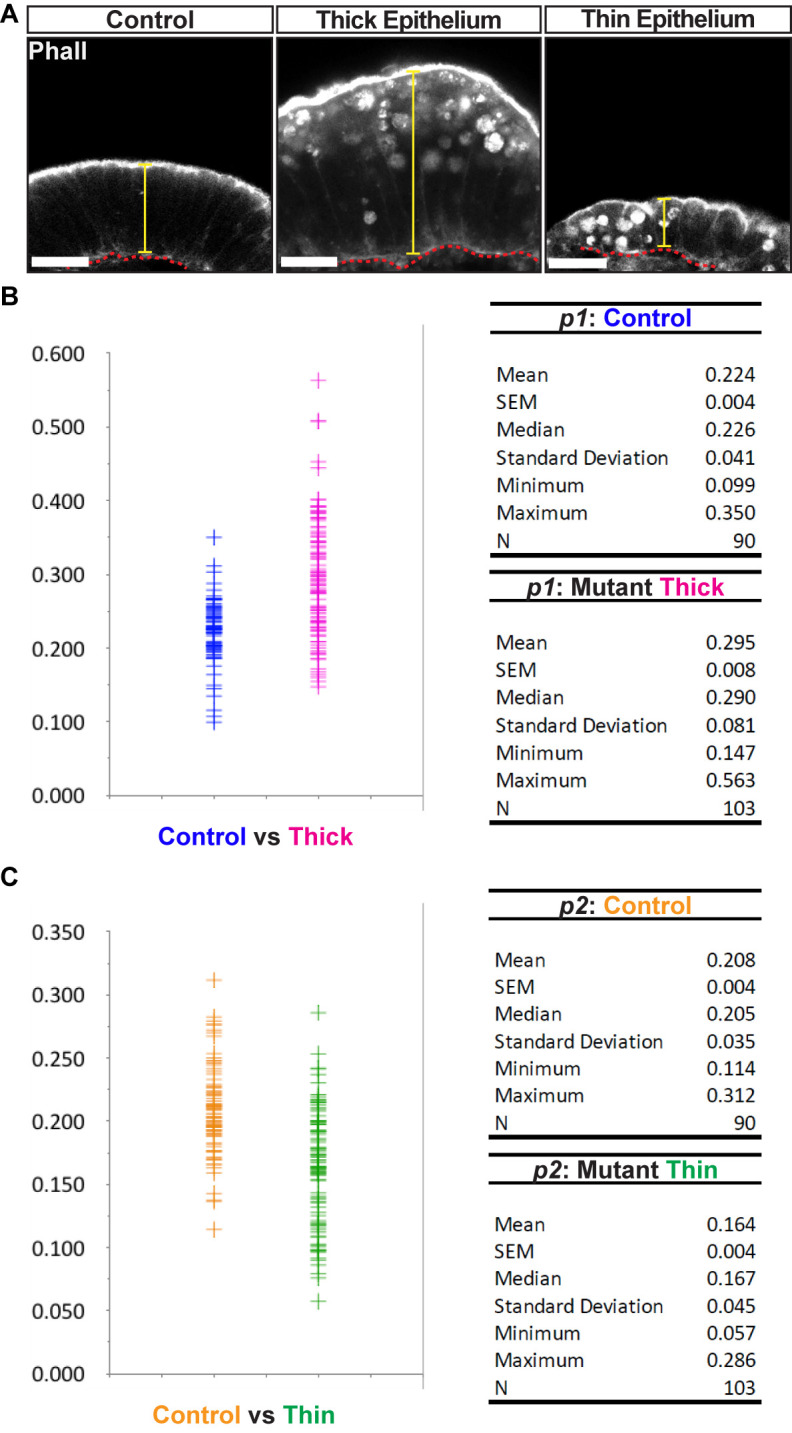
Different ectodermal thickness observed in CRISPR/Cas9 mutants. (**A**) Control and affected (thick and thin) epithelia were aligned at the base of the ectoderm (red line) for better visualization. Morphology is shown by Phalloidin staining. Yellow line depicts different cell length (thickness). (**B**) Dot plot displaying the range of phenotypes observed in thickness (*p1* = cell length/embryonic diameter) of the mutant thick epithelium compared to controls. Numerical data are displayed at the right. Mutant thick *p1* was larger than control *p1* (p-value=8.14E-11). (**C**) Dot plot displaying the range of phenotypes observed in thickness (*p2* = cell length/embryonic diameter) of the mutant thin epithelium compared to controls. Numerical data are shown at the right. Mutant thin *p2* was smaller than control *p2* (p-value=3.00E-11). The observed changes may be an abnormality caused by the loss of epithelial homeostasis. For detail on the measurements, see also Figure 3—figure supplement 7. Values and statistics can be found in Figure 3—figure supplement 3—source data 1. 10.7554/eLife.36740.011Figure 3—figure supplement 3—source data 1.Measures and statistical analyses related to Figure 3—figure supplement 3.

**Figure 3—figure supplement 4.**
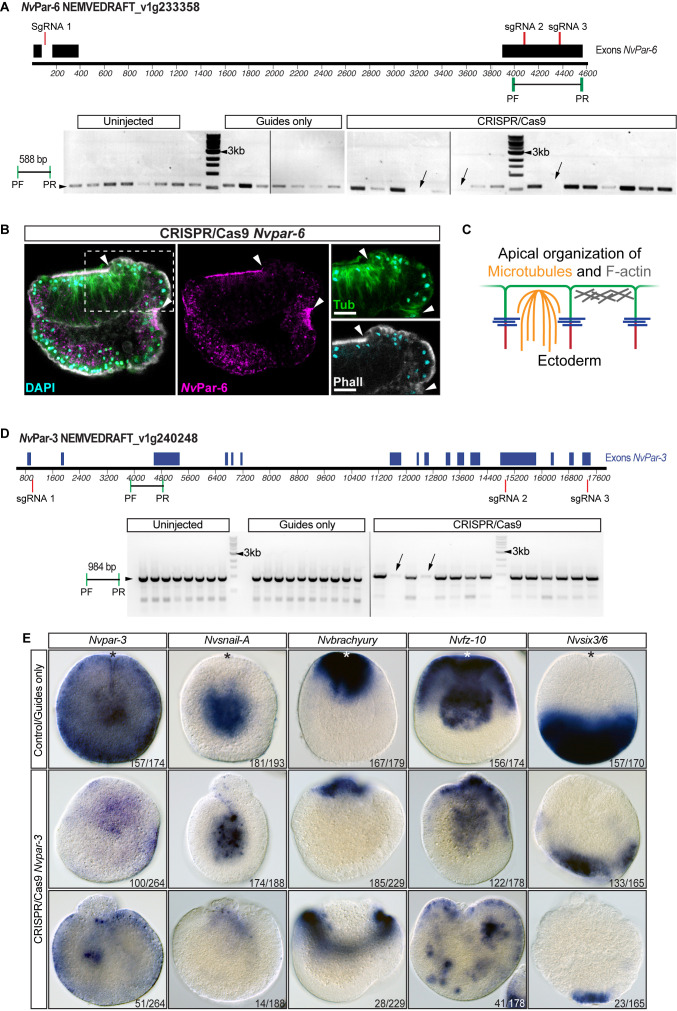
CRISPR/Cas9 mediated mutagenesis of *Nvpar-6* and *Nvpar-3.* (**A**) The position of the sgRNAs (red) and primers (green) used for the PCR assay are shown on the diagram depicting the genomic sequence of *Nvpar-6*. Note the absence of fragments of *Nvpar-6* (arrow) resulting from CRISPR/Cas9 mediated mutagenesis. The presence of other bands suggests mosaicism. Black rectangles correspond to *Nvpar-6* exon. PF: primer forward. PF: primer reverse. See also Figure 3—figure supplement 4—source data 1. (**B**) Immunofluorescent staining for cytoskeleton and *Nv*Par-6 in CRISPR/Cas9 *Nvpar-6* knock-out embryos (mosaic phenotype). Arrowheads indicate the absence of *Nv*Par-6. High magnification images are shown on the right. The cytoskeleton is apically organized only where *Nv*Par-6 is apically localized. (**C**) Diagram depicts the role of aPKC/Par complex (green) on cytoskeleton. (**D**) The position of the sgRNAs (red) and primers (green) used for the PCR assay are shown on the diagram depicting the genomic sequence of *Nvpar-3*. Note the absence/truncation of fragments of *Nvpar-3* (arrow) resulting from CRISPR/Cas9 mediated mutagenesis. The presence of other bands suggests mosaicism as shown in Figure 3—figure supplement 1E. Blue rectangles correspond to *Nvpar-3* exon. PF: primer forward. PF: primer reverse. See also Figure 3—figure supplement 4—source data 1. (**E**) In situ hybridization of *Nvpar-3* knockout embryos (Cas9 and gRNAs) compared with control embryos at 40 hpf. The disruption of the *N. vectensis* Par/aPKC complex modified the morphology but did not modify the cell-fate specification of endomesodermal cells. Lower panel for *Nvfz-10* may represent an extreme case of mesenchymal-like endomesoderm. 10.7554/eLife.36740.013Figure 3—figure supplement 4—source data 2.Full height of the blots shown in Figure 3—figure supplement 4.White boxes correspond to the sections reported in Figure 3—figure supplement 4.

**Figure 3—figure supplement 5.**
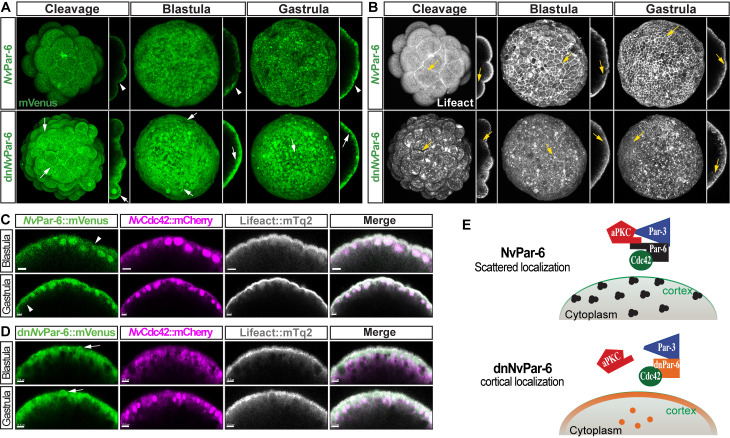
In vivo localization of dn*Nv*Par-6 protein at different embryonic stages. Images of the whole embryo correspond to a 3D reconstruction from a z-stack series. Side panels are a single optical section from the z-stack series. An aboral view is shown for all gastrula stages. Yellow arrows indicate the apico-lateral cortex labeled with Lifeact::mTq2. Scale bars: 10 µm. (**A**) In vivo localization of *Nv*Par-6::mVenus and dn*Nv*Par-6::mVenus at cleavage, blastula, and gastrula stages. *Nv*Par-6::mVenus distributes uniformly at the apical region of the cell but displays a scattered pattern. However, dn*Nv*Par-6::mVenus displays stronger cortical localization due to its interaction with *Nv*Cdc42 at the apical and apico-lateral cortex of the cells. This was confirmed by their co-expression with *Nv*Cdc42::mCherry. White arrowheads indicate the scattered localization of *Nv*Par-6::mVenus. White arrows indicate the cortical and stronger localization of dn*Nv*Par-6::mVenus. (**B**) In vivo localization of Lifeact::mTq2 in *Nv*Par-6::mVenus and dn*Nv*Par-6::mVenus expressing embryos shown in (**A**) at cleavage, blastula, and gastrula stages. The actin cytoskeleton was also affected by the overexpression of dn*Nv*Par-6::mVenus. (**C**) In vivo co-distribution of *Nv*Cdc42::mCherry with *Nv*Par-6::mVenus at blastula and gastrula stages. (**D**) In vivo co-distribution of *Nv*Cdc42::mCherry with dn*Nv*Par-6::mVenus at blastula and gastrula stages. *Nv*Cdc42::mCherry localization is also affected when dn*Nv*Par-6::mVenus is overexpressed. (**E**) Graphical summary of the observed results for *Nv*Par-6::mVenus and dn*Nv*Par-6::mVenus.

**Figure 3—figure supplement 6.**
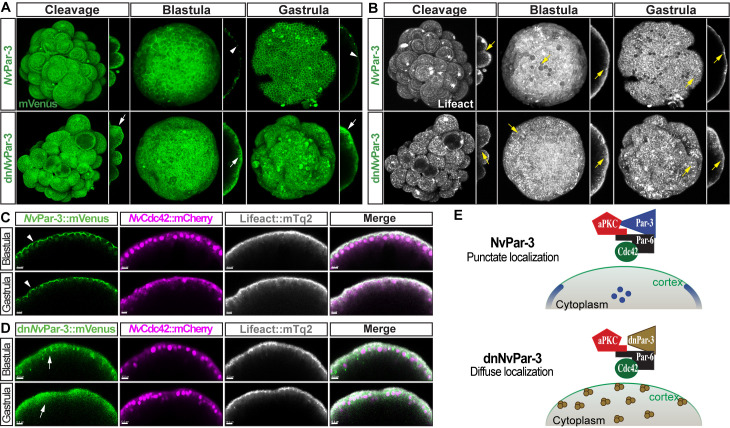
In vivo localization of dn*Nv*Par-3 protein at different embryonic stages. Images of the whole embryo correspond to a 3D reconstruction from a z-stack series. Side panels are a single optical section from the z-stack series. An aboral view is shown for all gastrula stages. Yellow arrows indicate the apico-lateral cortex labeled with Lifeact::mTq2. Scale bars: 10 µm. (**A**) In vivo localization of *Nv*Par-3::mVenus and dn*Nv*Par-3::mVenus at cleavage, blastula, and gastrula stages. dn*Nv*Par-3 displays broader localization, resembling the localization of *Nv*Par-6 and indicating its release from AJs (compare with *Nv*Par-3). White arrowheads indicate the punctate localization of *Nv*Par-3::mVenus. White arrows indicate the broader localization of dn*Nv*Par-3::mVenus. (**B**) In vivo localization of Lifeact::mTq2 in *Nv*Par-3::mVenus and dn*Nv*Par-3::mVenus expressing embryos shown in (**F**) at cleavage, blastula, and gastrula stages. The actin cytoskeleton was also affected by the overexpression of dn*Nv*Par-3::mVenus. (**C**) In vivo co-distribution of *Nv*Cdc42::mCherry with *Nv*Par-3::mVenus at blastula and gastrula stages. (**D**) In vivo co-distribution of *Nv*Cdc42::mCherry with dn*Nv*Par-3::mVenus at blastula and gastrula stages. *Nv*Cdc42::mCherry localization is not affected when dn*Nv*Par-3::mVenus is overexpressed. (**E**) Graphical summary of the observed results for *Nv*Par-3::mVenus and dn*Nv*Par-3::mVenus.

**Figure 3—figure supplement 7.**
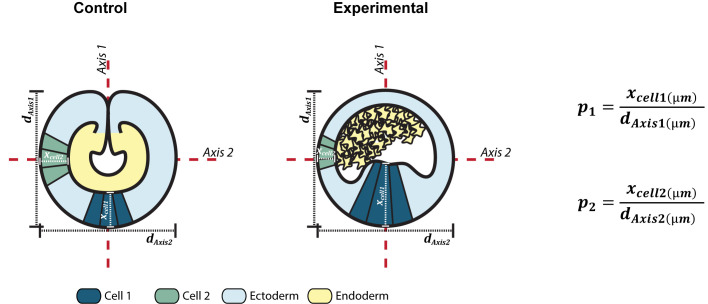
Morphometric measurements methodology. Schematic representation of the morphometric measurements used to compare the epithelial thickness between different treatments. In control embryos, two perpendicular axes were determined: Axis 1 corresponds to the axis in parallel with the Oral-Aboral axis of the embryo. Axis 2 was established as the axis perpendicular to the Axis 1. For experimental embryos, cells of the ‘mesenchymal endoderm’ were asymmetrically distributed inside the lumen of the embryo and the ectoderm presented thickened and thinned epithelium. Axis 1 was defined as the axis parallel to the thickened epithelium and the Axis 2 as the axis parallel to the thinned epithelium. ‘*daxis1*’ and ‘*daxis2*’ correspond to the diameter (d; µm) of the embryo parallel to the Axis 1 and Axis 2, respectively. ‘*xcell1*’ and ‘*xcell2*’ represent the average values for the apicobasal (**A–B**) length (µm) of the cells (epithelial thickness) in parallel to the Axis 1 and Axis 2, respectively. Mathematical calculation of epithelial thickness: In order to compare differences in the thickness of ectodermal epithelium between different treatments, ‘*xcell1*’ and ‘*xcell2*’ were normalized by the diameter’ *daxis1*’ and ‘*daxis2*’, respectively, giving a proportion (p). In mutant embryos, A1 and A2 were not necessarily related to the O/A axis. Thus, first, we assessed if there were differences in the epithelial thickness between the ectodermal epithelium of both axes. In control embryos, the aboral ectodermal epithelium is parallel with A1 and was significantly thicker than the lateral ectodermal epithelium parallel with A2 (p_1control_> p_2control_; p-value=0.00036).

**Figure 3—video 1.** Related to Figure 3E. 3-D rendering of CRISPR phenotype. Tubulin and DAPI are shown in green and magenta, respectively. See also Figure 3—animation 1.

**Figure 4.**
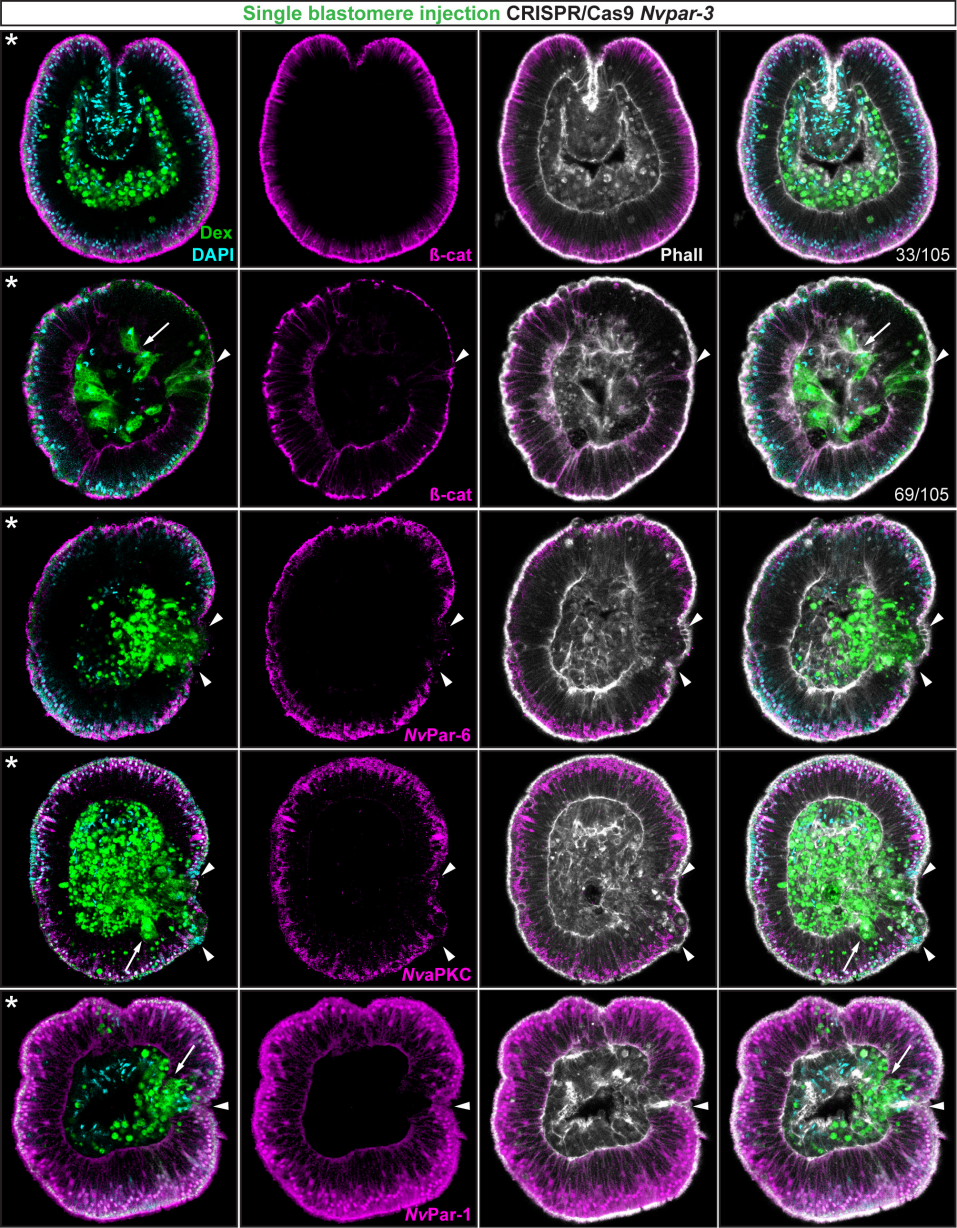
Ectodermal- but not endomesodermal-lineages are affected by single injected-blastomere CRISPR/Cas9 *Nvpar-3* knock-outs. IFS for ß-catenin (ß-cat), *Nv*Par-6, *Nv*aPKC, and *Nv*Par-1 in single injected-blastomere CRISPR/Cas9 *Nv*Par-3 knock-outs at 40 hpf. Streptavidin-Biotin TxRed Dextran (Dex) is shown in green. Arrowheads indicate the absence of the protein and disrupted epithelium. Arrows indicate bottle-like shape cells. * indicate the orientation of the site of gastrulation. See also Figure 4—figure supplements 1 and 2, and Figure 4—video 1. Morphology is shown by DAPI and Phalloidin. All images are single optical sections from the z-stack confocal series.

We further tested this hypothesis by using genome editing by CRISPR/Cas9 targeting *Nvpar-6* and *Nvpar-3* genes (Figure 3D). We did not observe any effects on the embryo until 36 hpf at 16°C (late blastula stage), indicating the activity of maternally loaded proteins up until that stage. When *Nv*Par-6 and *Nv*Par-3 are mutated, the ectodermal epithelium loses its integrity, presenting changes in thickness (Figure 3—figure supplement 1B and Figure 3—figure supplement 3), and interestingly, the endomesoderm (which does not express these proteins) generates cells with mesenchymal-like morphotypes that are never normally seen in this species (Figure 1D and E). In *Nvpar-6* and *Nvpar-3* mutant embryos, we also observed the disruption of microtubules and actin cytoskeleton (Figure 3—figure supplement 4B), and AJs (visualized with the ß-catenin antibody in Figure 4) that confirms our previous observations of their role in regulating ectodermal cell polarity. Although it was difficult to dissect significant changes in the expression of germ layer markers (e.g. *Nvbra*, *Nvsnail*, *NvSix3/6*, and *Nvfz10*) from the morphological changes associated with epithelial integrity when these genes were disrupted (Figure 3—figure supplement 4E), it is clear that the primary defect in NvPar3 KO were aspects of cell adhesion and not cell type specification. Similar results were obtained when we overexpressed the mRNA encoding for a dominant negative version *Nv*Par-6 (dn*Nv*Par-6) and *Nv*Par-3 (dn*Nv*Par-3) into *N. vectensis* eggs (Figure 3—figure supplement 1 and Figure 3—figure supplements 5 and 6). However, dominant negative effects on the injected embryos were observed at earlier stages (10–12 hpf) than the CRISPR/Cas9 mutants (zygotic expression) because the mutant proteins compete with the wild type proteins (maternally loaded). Hence, in these experiments, embryonic lethality (90%) and cell death were higher.

**Figure 4—figure supplement 1.**
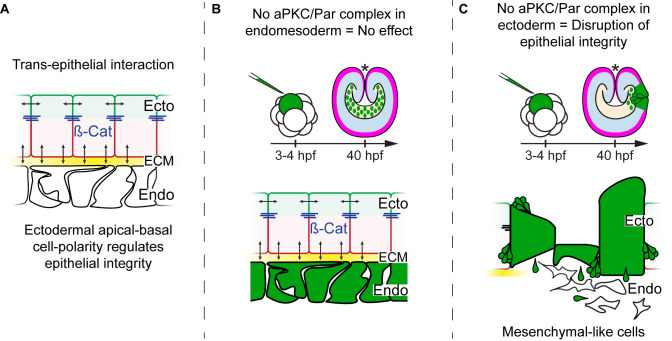
Diagram depicting the hypothesis addressed in Figure 4. CRISPR/Cas9 *Nv*Par-3 knock-outs in single blastomere injections suggest that the apicobasal polarity of ectodermal cells regulates trans-epithelially the integrity of endomesodermal epithelium. However, no effects are observed when endodermal cells were treated. The cell lineage derived from a single injected-blastomere is in green.

**Figure 4—figure supplement 2.**
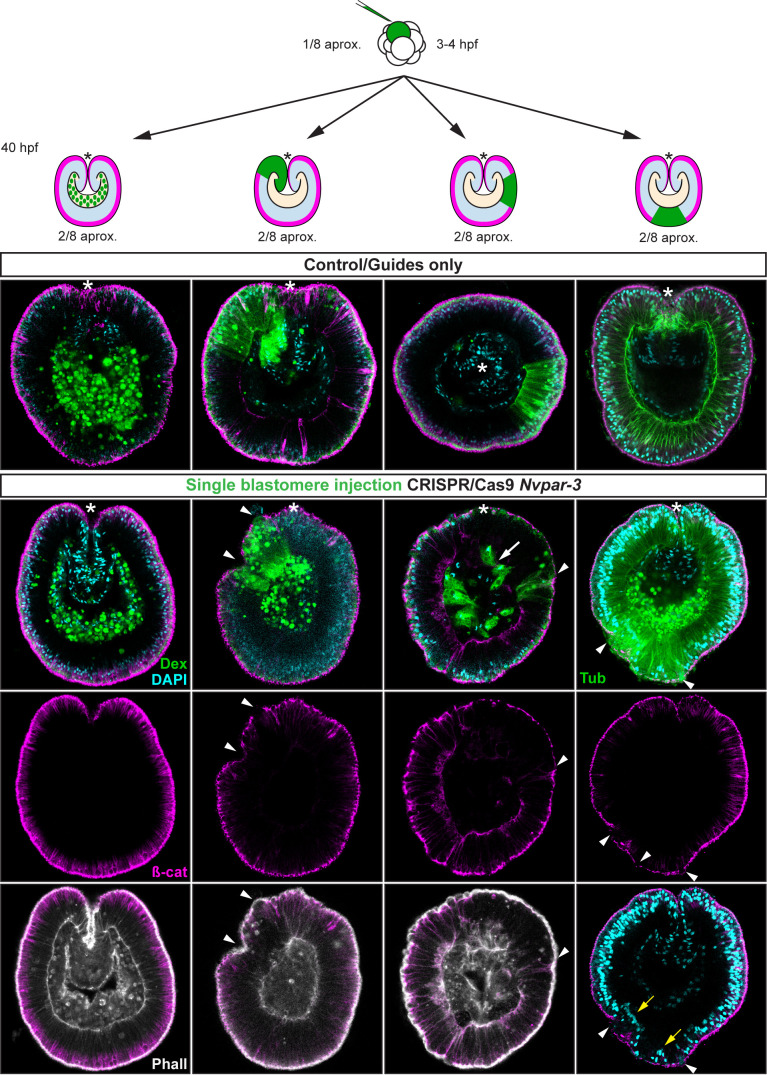
Immunofluorescent staining for ß-catenin (ß-cat) in single injected-blastomere control and CRISPR/Cas9 *Nvpar-3* knock-outs at 40 hpf. Morphology is shown by DAPI and Phalloidin staining. Streptavidin-Biotin TxRed Dextran (Dex) is shown in green. Arrowheads indicate the absence of the protein and disrupted epithelium. Arrows indicate bottle-like shape cells. Note the displacement of nuclei (yellow arrow) and changes in tubulin (Tub) staining. *: site of gastrulation is up.

**Figure 4—video 1.** z-stack series of CRISPR phenotype (ß-catenin panel). Streptavidin-Biotin TxRed Dextran (Dextran) and Phalloidin are shown in green and gray, respectively. Arrow (magenta) indicates an extruding cell. See also Figure 4—animation 1 and Figure 4—animation 2.

### The *Nv*aPKC/Par complex regulates transepithelial signaling

One surprising observation from the experiments described above show that the changes observed in the ectodermal and endodermal epithelium after disrupting *Nv*Par-6 and *Nv*Par-3 (Figure 3) suggests some sort of trans-epithelial regulation of cell–cell adhesion (most likely involving AJs) because these Par genes are not expressed in the endomesoderm. The polarizing activity of the aPKC/Par complex in the ectoderm is thus necessary to maintain the integrity of both ecto- and endodermal epithelia during cellular movements associated with gastrulation.

To assess whether the observed phenotypes on cell-cell adhesion are related to non-autonomous cell regulation (trans-epithelial interactions), we repeated the above experiments randomly injecting single blastomeres at 3–4 hpf (8–16 cell-stage) to make mutant clones in an otherwise wild type background (Figure 4). In these experiments, only the cell-lineage of the injected blastomere would be affected and would exhibit defective cell-cell adhesion in an otherwise undisturbed wild-type background. If endomesodermal cells derived from an injected blastomere display fibroblast/mesenchymal cell morphology, it would indicate that the organization of the endomesodermal epithelium is not dependent on the ectoderm but, rather, an intrinsic cell-autonomous activity of the aPKC/Par complex (Figure 4—figure supplement 1). Our results show that only ectodermal- but not endomesodermal-lineages are affected by these mutations (Figure 4 and Figure 4—figure supplement 2). Presumptive ectodermal cells derived from an injected blastomere fail to maintain AJs (and potentially SJs) and the resulting clone of epithelial cells loses its structural integrity inducing cell extrusion. In contrast, presumptive endomesodermal cells derived from an injected blastomere develop into a normal endomesodermal epithelium (Figure 3F). Our results complement the work of (Kirillova et al., 2018) and demonstrate that the proper cell-cell adhesion of the ectodermal layer somehow regulates trans-epithelially the integrity of the endomesodermal layer. This regulation may maintain the tension between cells during invagination at gastrula stages, or, in conjunction with the extracellular matrix (ECM) and basal cues, it may influence signaling patterns necessary to organize epithelial layers during *N. vectensis* embryogenesis.

### Interaction between the *Nv*aPKC/Par complex and the canonical Wnt signaling pathway

#### *Nv*aPKC/Par complex regulates ß-catenin localization

**Figure 5.**
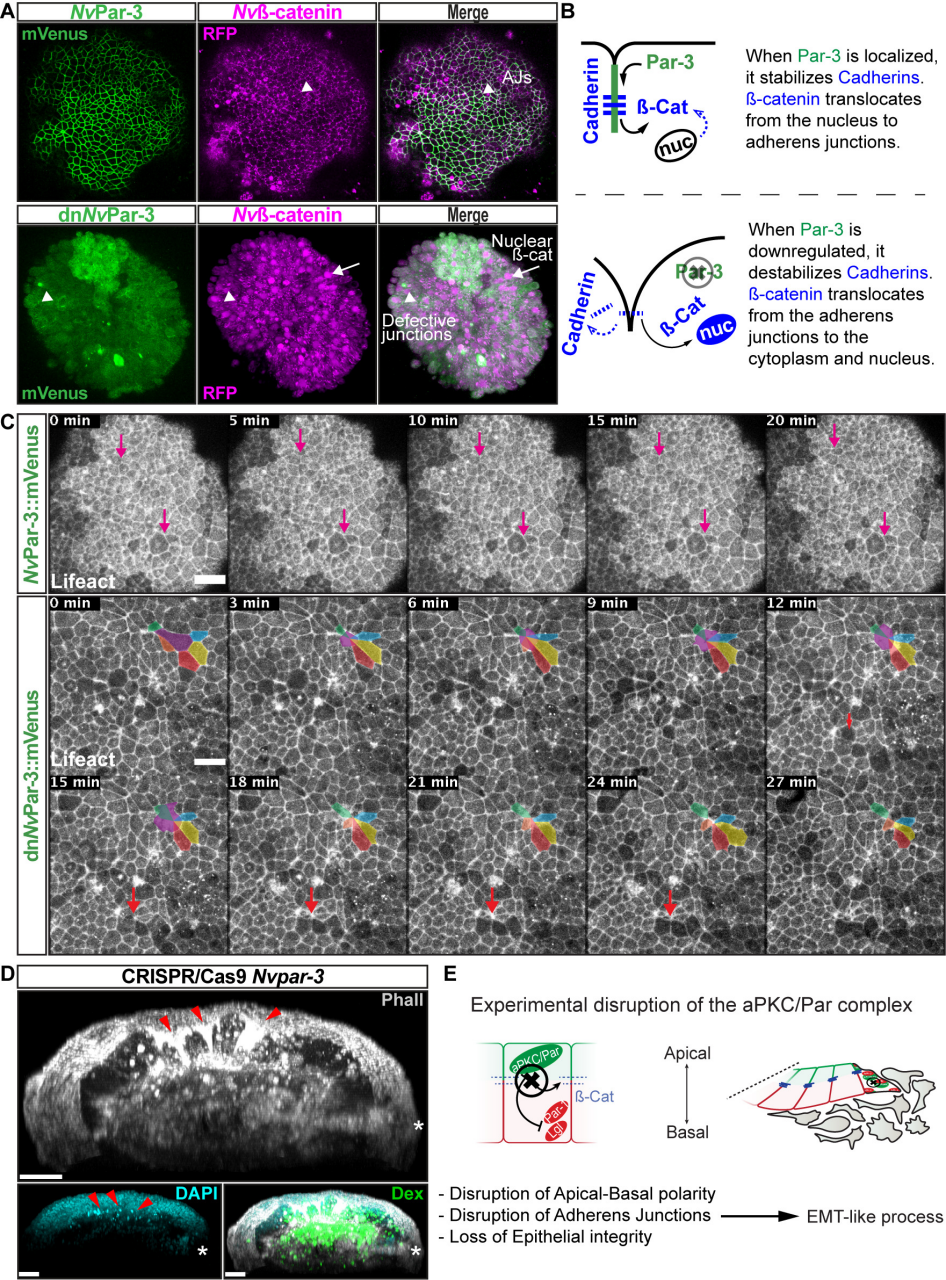
*Nv*aPKC/Par complex regulates ß-catenin localization and cell attachment. (**A**) In vivo co-localization of *Nv*Par-3venus co-injected with *Nv*ß-cateninRFP, and dn*Nv*Par-3venus co-injected with *Nv*ß-cateninRFP. Arrowheads indicate junctions (AJs). Arrows indicate nuclear ß-catenin. (**B**) Diagram of the suggested interpretation for A. (**C**) In vivo time series of ectodermal epithelial layers of embryos injected with *Nv*Par-3venus and dn*Nv*Par-3venus mRNA demonstrating epithelial delamination in the absence of functional *Nv*Par3. Lifeact::mTq2 mRNA was co-injected to visualize cell boundaries. Pink arrows indicate the absence cell detachments. A subset of cells was artificially colored. The purple cell detaches from the epithelium and the red arrow indicates a second celldetachment. See also Figure 5—video 1. (**D**) IFS of an embryo in which a single blastomere was injected with *Nv*Par-3 guide RNAs and Cas9 and green dextran. Red arrowheads indicate the apical constriction and delamination of ectodermal cells in the mutated clone of cells. Note the different layers of nuclei stained with DAPI. Asterisks indicate the site of gastrulation. (**E**) Diagram of the suggested interpretation for D. All images are 3D reconstructions from a z-stack confocal series. All scale bars, 20 µm.

In bilaterians, AJs recruit members of the aPKC/Par complex and the direct interaction between Par-3 and aPKC/Par-6 is required for the maintenance and maturation of AJs (Ohno et al., 2015; Ragkousi et al., 2017). AJs are characterized by the binding between cadherins and ß-catenin: cadherins sequester ß-catenin from the cytoplasm to the cortex, making it unavailable for nuclear signaling and endomesoderm specification (Wikramanayake et al., 2003; Kumburegama et al., 201124,41. Therefore, using ß-catenin as a marker for AJs, we separately co-injected *Nv*Par-3::mVenus or a mutated dn*Nv*Par-3::mVenus, with *Nv*ß-catenin::RFP into uncleaved zygotes. We observed cortical co-localization of *Nv*Par-3 and *Nv*ß-catenin at the cell boundaries in the ectodermal epithelium of embryos co-injected with *Nv*Par-3::mVenus and *Nv*ß-catenin::RFP (Figure 5A). However, in embryos co-injected with *Nv*ß-catenin::RFP and dn*Nv*Par-3::mVenus, we observed an alteration of the sub-cellular expression of *Nv*ß-catenin::RFP in all cells due to the translocation of ß-catenin from the cortical AJs into cell nuclei (Figure 5A).

**Figure 5—figure supplement 1.**
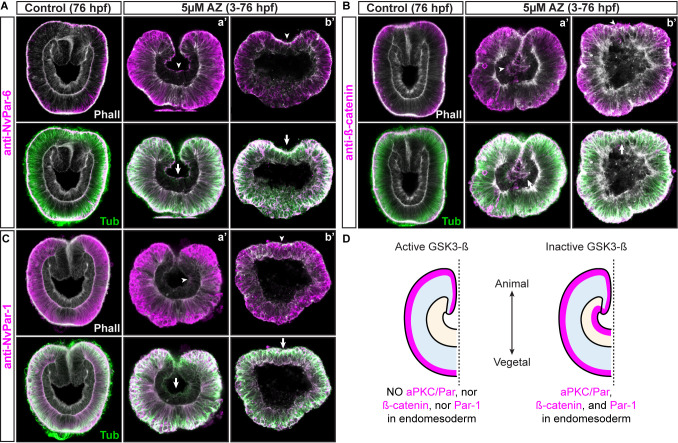
Apical junctions are regulated by GSK-3ß in epithelial cells during gastrulation in *N. vectensis* embryos. (**A**) IFS for *Nv*Par-6 in AZ-treated embryos. (**B**) IFS for ß-catenin in AZ-treated embryos. (**C**) IFS for *Nv*Par-1 in AZ-treated embryos. (**D**) Graphical summary of the observed results. Par-6, ß-catenin, and Par-1 were detected at the apical cortex (arrowheads) of the endomesodermal epithelium of AZ-treated embryos, but not in control embryos. Arrows indicated the stabilization of microtubules at the apical cortex of endomesodermal cells. Two distinct phenotypes were observed after AZ treatment: (**a’**) gastrulation without elongation 27% (27/100), (**b’**) no invagination of the endomesoderm 13% (13/100).

**Figure 5—video 1.** Related to Figure 5C. EMT-like process occurs in dn*Nv*Par-3::mVenus expressing cells. Lifeact::mTq2 is shown in grey. Arrows indicate the apical constriction of detaching cells. Scale bars:10 µm

In addition, results from *N. vectensis* embryos treated with 5 µm 1-azakenpaullone (AZ; an inhibitor of GSK-3ß and a canonical Wnt agonist) suggest that GSK-3ß stabilizes AJs of epithelial cells in *N. vectensis* embryos (Figure 5—figure supplement 1). We observed an expansion of the expression domain of Par-6 (Figure 5—figure supplement 1) and a stabilization of AJs (labeled with ß-catenin in Figure 5—figure supplement 1) in endomesodermal cells of treated embryos, which was never observed in control embryos.

Interestingly, the association between the nuclearization of ß-catenin (canonical Wnt signaling pathway) and the Par system has been poorly studied. Two studies, one in *Drosophila* (Sun et al., 2001) and the another in *Xenopus* (Ossipova et al., 2005) embryos, have shown by immunoblotting that the kinase Par-1 is associated with Dishevelled protein and might act as a positive regulator of Wnt signaling. Here, we show *in vivo* embryonic evidence suggesting that *Nv*Par-3 (whose cortical localization is normally inhibited by Par-1) recruits *Nv*ß-catenin protein and stabilizes its localization at the apico-lateral cortex of ectodermal cells through the formation of AJs. Furthermore, the putative disassembly of the aPKC/Par complex induced by dn*Nv*Par-3 overexpression, induces the nuclearization of *Nv*ß-catenin protein (Figure 5A) due to its cytosolic availability caused by AJs disruption. Strikingly, we also observed the extrusion of individual cells from the ectodermal epithelium of dn*Nv*Par-3 treated-embryos (Figure 5C) and single injected-blastomere CRISPR/Cas9 *Nv*Par-3 knock-out (Figure 5D). This suggests that these treatments induce EMT-like processes, not observed under control conditions (Figure 5C).

Thus, our data suggest that preexisting mechanisms downstream to the induction of EMT may have been redeployed to segregate layers during the evolution to bilaterians. Bringing the question whether or not endomesodermal genes would induce similar effects when they are expressed in *N. vectensis* embryos.

We have recently showed that *Nvbrachyury* regulates apicobasal polarity of epithelial cells in *N. vectensis* embryos (Servetnick et al., 2017). We, therefore, examined the role of *Nvsnail* genes on the localization of ß-catenin, components of the Par system, and the stabilization of AJs. Our hypothesis is that expression of *N. vectensis snail* genes would destabilize AJs and induce the nuclearization of ß-catenin in ectodermal epithelial cells.

### *Nvsnail* genes induce the translocation of *Nv*ß-catenin from AJs to the cytoplasm

**Figure 6.**
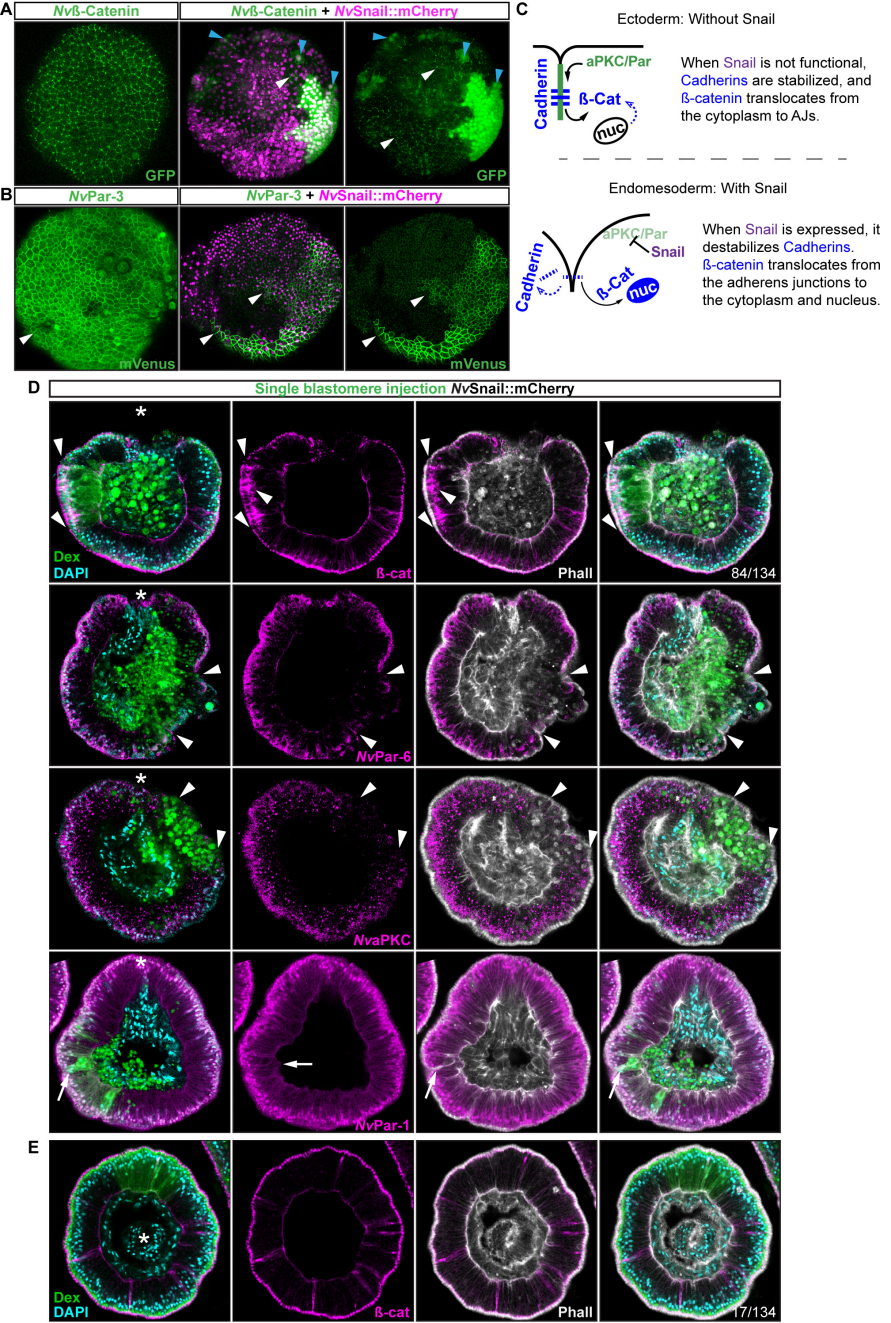
*Nvsnail* genes induce the translocation of *Nv*ß-catenin and the disruption of epithelial integrity. (**A**) *in vivo* localization of *Nv*ß-cateninGFP co-injected with both *Nv*Snail-A::mCherry and *Nv*Snail-B::mCherry mRNA together in zygotes at 40 hpf. White arrowheads indicate AJs. Patched patterns of cytosolic and nuclear ß-catenin (Blue arrowheads) were observed. (**B**) *in vivo* localization of *Nv*Par-3::mVenus co-injected with both *Nv*Snail-A::mCherry and *Nv*Snail-B::mCherry mRNA together at 40 hpf. Patched patterns of AJs (White arrowheads) were observed. (**C**) Diagram depicts the suggested interpretation for A and B. (**D**) IFS for ß-catenin (ß-cat), *Nv*Par-6, *Nv*aPKC, and *Nv*Par-1 in embryos at 40 hpf where *Nv*Snail-A::mCherry and *Nv*Snail-B::mCherry mRNA were overexpressed together into a single ectodermal blastomere lineage (followed by green Streptavidin-TxRed Dextran (Dex). Arrowheads indicate the absence of the protein, cytosolic ß-cat, and disrupted epithelium. Arrows indicate bottle-like shape cells. (**E**) IFS for ß-cat in embryos at 40 hpf where *Nv*Snail-A::mCherry and *Nv*Snail-B::mCherry mRNA were overexpressed together into a single blastomere lineage and no affects were observed. See also Figure 6—figure supplement 1, Figure 6—video 1, and Figure 6—video 2. *site of gastrulation. Morphology is shown by DAPI and Phall IFS. Except from 6A and 6B, all images are single optical sections from the z-stack confocal series. (**A**) and (**B**) are 3D reconstructions from a z-stack confocal series.

*N. vectensis* has two *snail* genes, *Nvsnail-A* and *Nvsnail-B*, which are both expressed in the endomesodermal plate prior to and throughout the gastrulation process, and which define the boundary between gastrodermis and ectodermal pharynx (Magie et al., 2007; Röttinger et al., 2012; Amiel et al., 2017). To determine the role of *Nvsnail* genes on ß-catenin nuclearization, we co-injected the mRNA of *Nv*Snail-A::mCherry, *Nv*Snail-B::mCherry, and *Nv*ß-catenin::GFP into uncleaved eggs. The overexpression of both proteins *Nv*Snail-A::mCherry and *Nv*Snail-B::mCherry together induce the ectopic translocation of *Nv*ß-catenin::GFP to the nuclei of ectodermal cells (Figure 6A). This treatment also delocalizes *Nv*Par-3 from the cell cortex when both *Nv*Snail::mCherry proteins are co-expressed with *Nv*Par-3::mVenus (Figure 6B).

**Figure 6—figure supplement 1.**
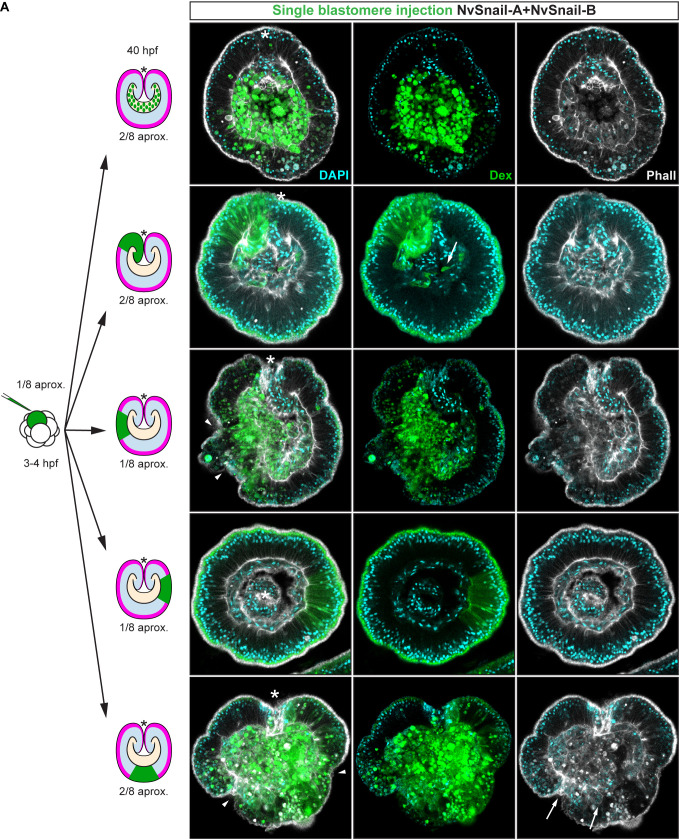
*Nvsnail-A and Nvsnail-B* together regulate epithelial integrity. Immunofluorescent staining of embryos at 40 hpf where *Nv*Snail-A::mCherry and *Nv*Snail-B::mCherry mRNA were overexpressed together into a single blastomere lineage (followed by green Streptavidin-TxRed Dextran (Dex). No affects were observed in approximately 1/8 of the injected embryos. Arrowheads indicate the disrupted epithelium. Arrows indicate bottle-like shape cells. *site of gastrulation. Morphology is shown by DAPI and Phall IFS. All images are single optical sections from the z-stack confocal series.

**Figure 6—video 1.** Related to Figure 6E. z-stack series of mRNA overexpression phenotype (NvPar-6 panel). Streptavidin-Biotin TxRed Dextran (Dextran) and Phalloidin are shown in green and gray, respectively. Arrow (magenta) indicates an extruding cell. See also Figure 6—animation 1.

**Figure 6—video 2.** Related to Figure 6E. z-stack series of mRNA overexpression phenotype (NvPar-1 panel). Streptavidin-Biotin TxRed Dextran (Dextran) and Phalloidin are shown in green and gray, respectively. Arrow (magenta) indicates an extruding cell. See also Figure 6—animation 2.

To determine the role of *Nvsnail* genes on cell adhesion/epithelial polarity, we randomly injected single blastomeres at the 8–32 cell-stage with mRNA from both *Nv*Snail-A::mCherry and *Nv*Snail-B::mCherry together. The fluorescent dextran that was co-injected with the mRNAs could be used to detect the clones where the over-expression of the co-injected mRNAs occurred in a ‘wild-type’ background (Figure 6D). Similar to the *Nvpar-3* knock-out (Figure 4), the expression of *Nvsnail* genes is sufficient to induce the degradation of Par proteins and AJs (ß-catenin) from the ectoderm and disrupts its epithelial integrity; however, nuclear ß-catenin was not observed under these treatments (Figure 6D). Thus, nuclear *Nv*ß-catenin::GFP observed *in vivo* when we overexpressed *Nv*Snail proteins (Figure 6A) is a consequence of the high cytosolic availability generated by its ectopic overexpression and release from AJs.

Interestingly, not every ectodermal cell was affected by these treatments even though all of the cells expressed the injected mRNAs (Figure 6A and E, and Figure 6—figure supplement 1). This patched pattern suggests that the response to *Nvsnail* over-expression is spatially regulated. These results suggest that the role of *Nvsnail* genes on AJs and apicobasal cell polarity is constrained to the site of gastrulation in *N. vectensis* embryos under natural conditions, and that these genes may be required for gastrulation movements. Therefore, we predicted that ß-catenin (AJs) and Par proteins will be retained in the cells of the *N. vectensis* endomesodermal plate if both *Nvsnail* genes are disrupted.

### *Nvsnail* genes downregulate apicobasal cell polarity and AJs in the endomesodermal epithelium of *N. vectensis* embryos

The *snail* genes temporally down-regulate E-cadherin during mesoderm segregation and EMT in bilaterian animals (Lim and Thiery, 2012). As we have shown here, as well as in previous studies (Magie et al., 2007; Magie and Martindale, 2008), the cells comprising the endomesodermal plate lose their cell-cell adhesion during gastrulation in *N. vectensis* embryos. It may be possible that temporal regulation of endomesodermal patterning might act upon the AJs. Our data suggest that once gastrulation is complete and the pharynx forms, components of the Par system and the ß-catenin components of the AJs are degraded from both the cortex and cytoplasm of endomesodermal cells (Figure 1 and Figure 1—figure supplement 1). Hence, it could be possible that *Nvbrachyury* induces the disruption of apicobasal polarity (Servetnick et al., 2017), remnant AJs maintain the endomesodermal-plate cells together, and *Nvsnail* genes degrades and prevents the reassembly of AJs in the endomesoderm of *N. vectensis*.

**Figure 7.**
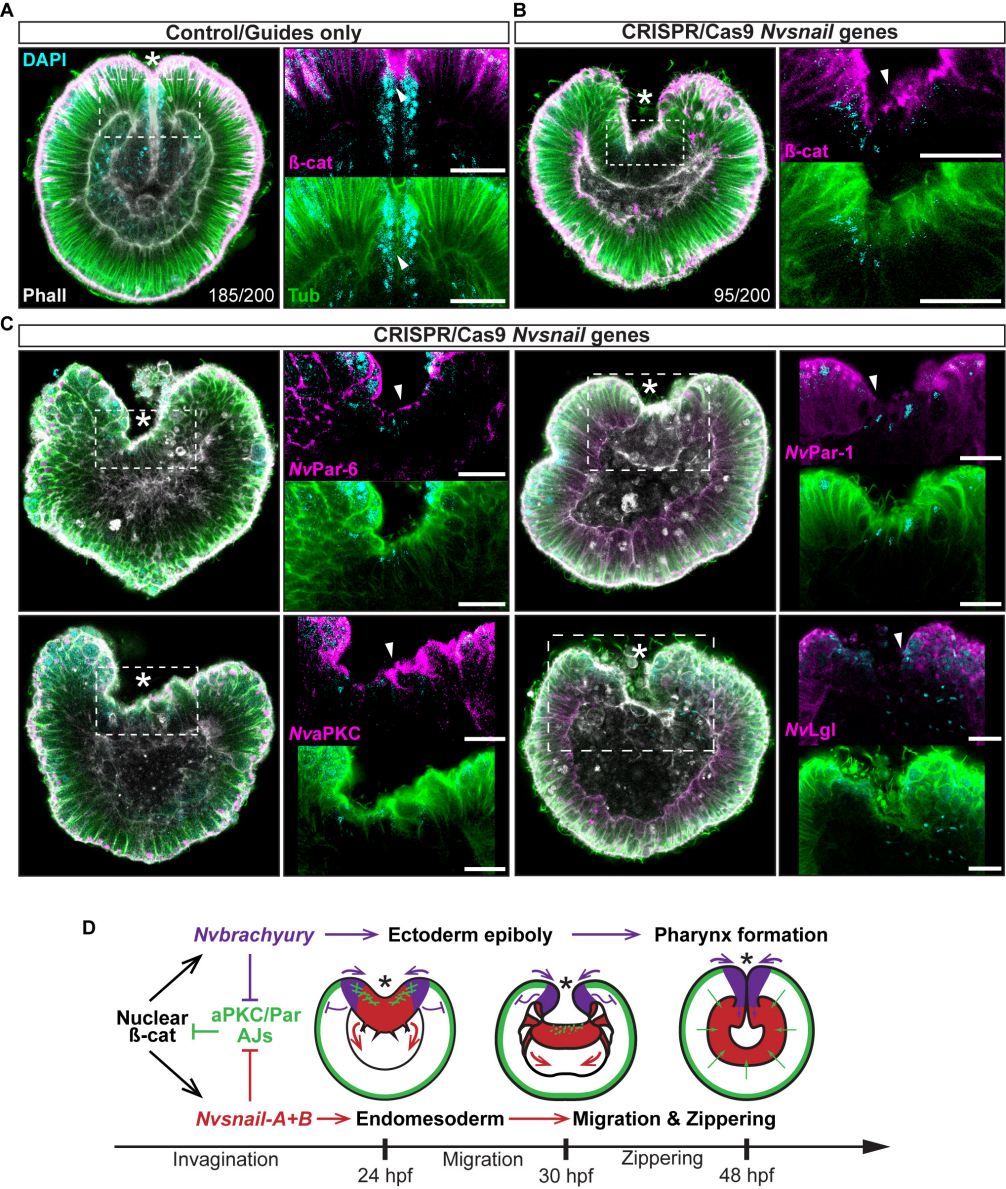
*Nvsnail* genes prevents the reassembly AJs and *Nv*aPKC/Par polarity allowing endomesodermal migration. (**A**) Embryo wide CRISPR/Cas9 knock-out guides controls show no effect neither on gastrulation nor AJs (ß-catenin) localization. (**B**) Embryo wide CRISPR/Cas9 knock-out for both *Nvsnail-A* and *Nvsnail-B* at 40 hpf showing that AJs are retained in presumptive endomesodermal region similar to ectodermal cells. High magnification images from boxed region (endomesodermal plate) are shown on the right. (**C**) Embryo wide CRISPR/Cas9 knock-out for both *Nvsnail-A* and *Nvsnail-B* at 40 hpf showing that *Nv*Par-6 and *Nv*aPKC proteins are retained in in presumptive endomesodermal region similar to ectodermal cells. *Nv*Par-1 and *Nv*Lgl were not detected in endomesodermal cells. High magnification images from boxed region (endomesodermal plate) are shown on the right. (**D**) Graphical summary of the observed results with previous published data (Servetnick et al., 2017). See also Figure 7—figure supplements 1 and 2. Morphology is shown by DAPI, Tubulin, and Phalloidin. All images are single optical sections from the z-stack confocal series. Arrowheads indicate protein localization. *site of gastrulation. All scale bars, 20 µm.

To address these issues, we used CRISPR/Cas9 knock-out of *Nvsnail-A* and *Nvsnail-B* genes together to inhibit zygotic function of these genes and investigate their role on the temporal regulation of AJs and cell polarity. In CRISPR/Cas9 mutants, the endomesodermal plate forms but it does not migrate further than its first invagination during gastrulation (Figure 7). Furthermore, AJs (labeled with ß-catenin) and apical Par proteins (labeled with anti*Nv*Par-6 and anti*Nv*aPKC) are retained at the apical cortex of the cells of the endomesodermal plate (Figure 7B and C and Figure 7—figure supplement 1). Surprisingly, *Nv*Par-1 and *Nv*Lgl were not detected in those cells (Figure 7C), suggesting that the degradation of these basolateral proteins precede or do not depend on the activity of the *Nvsnail* genes. This suggests that *Nvsnail* regulates apical cell-polarity, AJs turnover, and the migration (‘zippering’) but not the invagination of the endomesodermal plate during gastrulation of *N. vectensis* embryos (Figure 7D). Interestingly, the invagination of the endomesodermal plate (controlled by the Wnt/PCP pathway) is uncoupled from its specification in *N. vectensis* embryos (Kumburegama et al., 2011; Wijesena et al., 2011), which is consistent with our observations.

**Figure 7—figure supplement 1.**
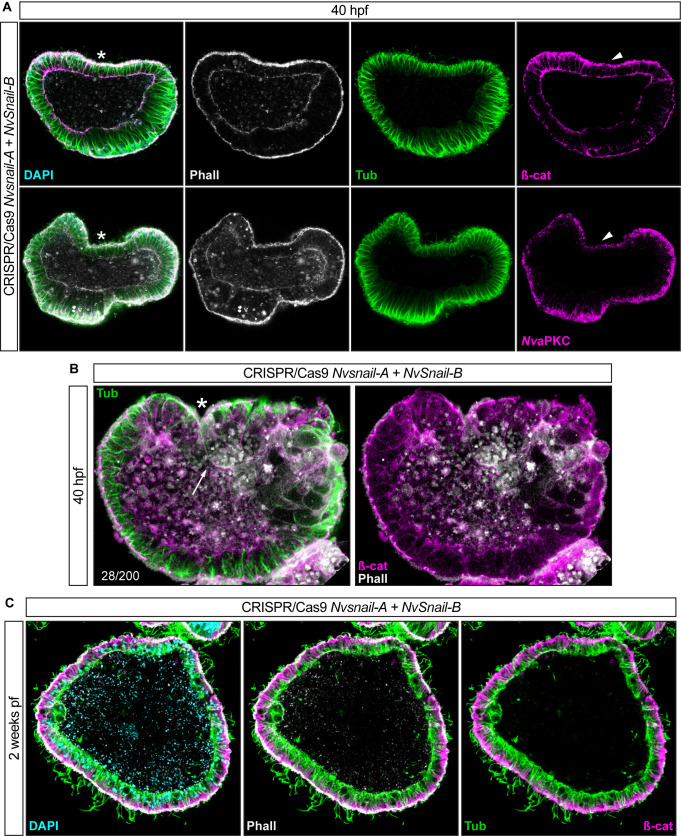
*Nvsnail-A and Nvsnail-B* together regulate AJs. *site of gastrulation. (**A**) Embryo wide CRISPR/Cas9 knock-out for both *Nvsnail-A* and *Nvsnail-B* at 40 hpf showing embryos that did not gastrulate and retain ß-catenin (ß-cat) and *Nv*aPKC at the apical cell-cortex of the endomesodermal plate (arrowheads). (**B**) Embryo wide CRISPR/Cas9 knock-out for both *Nvsnail-A* and *Nvsnail-B* at 40 hpf showing embryos that developed a pharynx (arrow) but not an organized endomesoderm. (**C**) Embryo wide CRISPR/Cas9 knock-out for both *Nvsnail-A* and *Nvsnail-B* at 2 weeks post fertilization (pf) showing embryos that did not develop a gastrodermal epithelium.

**Figure 7—figure supplement 2.**
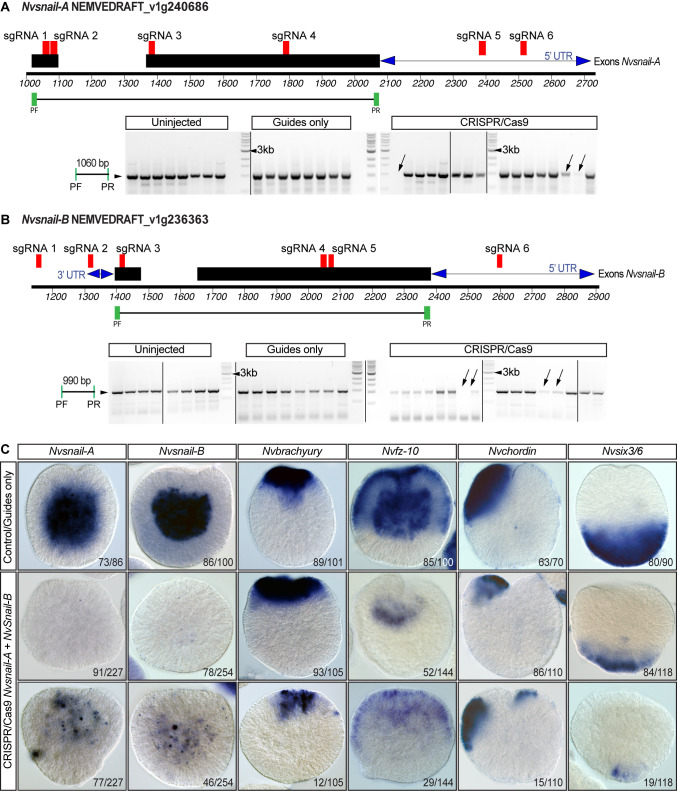
CRISPR/Cas9 mediated mutagenesis of *Nvsnail-A and Nvsnail-B.* (**A**) The position of the sgRNAs (red) and primers (green) used for the PCR assay are shown on the diagram depicting the genomic sequence of *Nvsnail-A*. Note the absence of fragments of *Nvsnail-A* (arrow) resulting from CRISPR/Cas9-mediated mutagenesis. The presence of other bands suggests mosaicism. Black rectangles correspond to *Nvsnail-A* exon. Blue double arrow depicts UTR regions. PF: primer forward. PF: primer reverse. See also Figure 7—figure supplement 2—source data 1. (**B**) The position of the sgRNAs (red) and primers (green) used for the PCR assay are shown on the diagram depicting the genomic sequence of *Nvsnail-B*. Note the absence of fragments of *Nvsnail-B* (arrow) resulting from CRISPR/Cas9 mediated mutagenesis. The presence of other bands suggests mosaicism as shown in (**C**). Black rectangles correspond to *Nvsnail-B* exon. Blue double arrow depicts UTR regions. PF: primer forward. PF: primer reverse. See also Figure 7—figure supplement 2—source data 1. (**C**) In situ hybridization of *Nvsnail-A+Nvsnail* B knockout embryos (Cas9 and gRNAs) compared with control embryos at 40 hpf. *Nvsnail-A+Nvsnail* B knockout embryos display no expression and mosaic expression of *Nvsnail-A* and *Nvsnail-B* but did not modify ectodermal specification (determined by the expression of *Nvbrachyury*, *Nvchordin*, and *Nvsix3/6* genes). KO embryos not shown displayed an expression pattern undistinguishable from controls. 10.7554/eLife.36740.037Figure 7—figure supplement 2—source data 1.Full height of the blots shown in Figure 7—figure supplement 2.White boxes correspond to the sections reported in Figure 7—figure supplement 2.

## Discussion

### AJs are down-regulated in mesoderm and neural crest of bilaterian animals

The segregation of different germ layers during embryogenesis of many bilaterian animals is carried out by similar cellular mechanisms. EMT is a shared mechanism utilized by mesoderm, neural crest cell (NCC), and tumorigenesis to delaminate cells in bilaterian animals (triploblastic animals). During EMT, the nuclearization of ß-catenin induces the expression of ‘endomesodermal’ genes like *brachyury* and *snail* (Acloque et al., 2009). The expression of these genes downregulates epithelial cadherins, disrupts apicobasal polarity (mediated by the aPKC/Par complex), disassembles AJs, and induces changes in cytoskeleton organization (Acloque et al., 2009; Lim and Thiery, 2012). A rearrangement of the actin-myosin cytoskeleton induces apical constriction of cells (generating a bottle-like shape), which detach from the epithelial sheet, break down the basal membrane, and invade a specific tissue as mesenchymal cells (Acloque et al., 2009; Lim and Thiery, 2012; Ohsawa et al., 2018).

Interestingly, mesoderm formation, tumorigenesis, and EMT have never been described as natural processes during *N. vectensis* (a diploblastic animal) embryogenesis. During *N. vectensis* gastrulation (Magie et al., 2007; Tamulonis et al., 2011), cells around the edge of the blastopore at the animal pole (which expresses *Nvbrachyury*) acquire a bottle-like shape by apical constriction, leading to epithelial buckling and the invagination of presumptive endomesoderm (which expresses *Nvsnail*). However, throughout this process the endomesoderm remains as a monolayer of epithelial cells and individual mesenchymal cells never detach and invade the blastocoel.

We have shown that by disrupting the aPKC/Par complex (apicobasal cell-polarity) in *N. vectensis* (Figures 3, 4 and 5), we are able to convert cells from the endomesodermal epithelium into mesenchymal-like cells, translocate *Nv*ß-catenin (Figure 5A), and emulate EMT-like processes (apical constriction and individual cell-detachments) in the ectodermal epithelium of *N. vectensis* treated-embryos (Figure 5C and D). These results demonstrate that the cnidarian *N. vectensis* possesses mechanisms necessary to segregate individual germ layers (e.g. mesoderm and NCC) described in bilaterians; however, they do not do it.

Our working hypothesis is that the *N. vectensis* embryo is composed of two independent morphogenetic modules that are integrated and organized by the pharynx (Figure 7D). The first observation is that the ectoderm, whose apicobasal polarity (and thus, AJs and epithelial integrity) is regulated by *Nvbrachyury* that promotes ectodermal epithelial morphogenesis and pharynx formation (Servetnick et al., 2017), and the second module is generated by endomesodermal differentiation and cell-movements that are regulated by *Nvsnail* genes. This is supported by the expression *Nvbrachyury* in *Nvsnail* knock-out embryos (Figure 7—figure supplement 2), and *Nvsnail* knock-out phenotypes where ectodermal pharynx develops normally but no clear endomesoderm is formed (Figure 7—figure supplement 1). Additional work is required to elucidate any differences in function between *Nvsnail-A* and *Nvsnail-B* genes, however, both modules are specified by nuclear ß-catenin (Röttinger et al., 2012), suggesting that the nuclear ß-catenin (maternal) shift from the animal pole in cnidarians to the vegetal pole in bilaterians is mechanistically plausible and sufficient to re-specify the site of gastrulation and germ-layers along the animal-vegetal axis during Metazoan evolution (Martindale and Lee, 2013; Lee et al., 2007).

### The dual identity and collective migration of the endomesodermal cells

**Figure 8.**
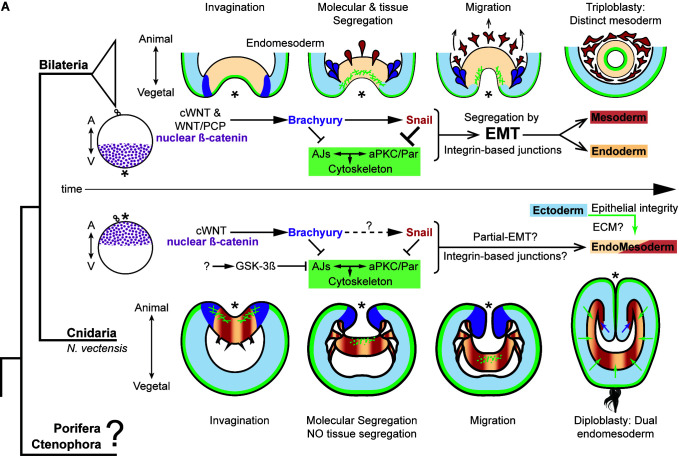
The differences between epithelial structure in ectoderm and endomesoderm in *N. vectensis* embryos are due to the lack of mechanisms to segregate a distinct mesoderm. Diagram depicting key cellular and molecular mechanisms involved during gastrulation of bilaterian and *N. vectensis* (a cnidarian) embryos. See also Figure 8—figure supplement 1.

Bilaterian-EMT has been a focus of study for decades as a mechanism to segregate different cell layers involved in a variety of different normal and pathological biological processes (Ohsawa et al., 2018; Nieto et al., 2016). This process appears to depend on the fine regulation of *snail* expression levels and their temporal activity. For example, during NCC migration, cells display ‘partial-EMT’ where cells remain attached to several neighboring cells but their apicobasal polarity and AJs are down-regulated, allowing collective-cell migration (Weng and Wieschaus, 2017; Nieto et al., 2016; Lee et al., 2006; Theveneau and Mayor, 2013; Ribeiro and Paredes, 2014). Our data suggest that ‘partial-EMT’ may be the mechanism by which the endomesodermal epithelium migrates into the blastocoel in *N. vectensis* embryos during normal gastrulation (Figure 8). In this scenario, upstream factors that regulate *snail* transcription may be critical for this process.

**Figure 8—figure supplement 1.**
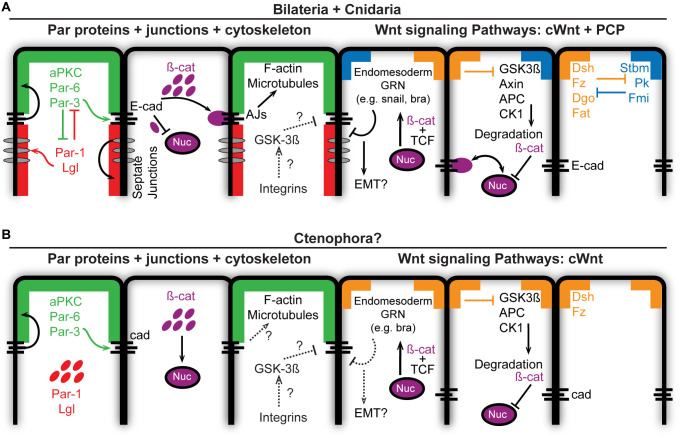
Suggested model for mesoderm specification in Metazoa. (**A**) Differential regulation of cell adhesion by the endomesoderm GRN is mediated by changes in cell polarity that regulate ß-catenin localization. These mechanisms emerged at the Bilateria + Cnidaria node. (**B**) Ctenophores do not possess a full complement of cell adhesion, cell-polarity, and endomesodermal GRN components present in the most common ancestor between Cnidaria and Bilateria. However, ctenophores possess a distinct mesoderm, suggesting the emergence of different mechanisms to segregate mesoderm in Metazoa.

In bilaterian animals, there are many other pathways in addition to the canonical Wnt pathway that activate *snail* transcription and induce the disruption of AJs and apicobasal cell polarity. For example, TGFß, BMP, NANOS, FGF, and MEK/ERK/ERG take on roles during the specification of mesoderm, NCC migration, tumorigenesis, and other EMT-related processes (Lim and Thiery, 2012; Nieto et al., 2016; Barrallo-Gimeno and Nieto, 2005). Concordantly in *N. vectensis* embryos, cells of the pharyngeal and endomesodermal tissues express components of all these pathways (Röttinger et al., 2012; Amiel et al., 2017; Extavour et al., 2005; Matus et al., 2006, 2007; Wijesena et al., 2017) that may modify their cellular characteristics. For example, one cadherin (*Nv*CDH2 Clarke et al., 2016: 1g244010), and kinases that modify tubulin and histones are differentially regulated between ecto- and endomesodermal epithelium (Wijesena et al., 2017).

In conclusion, *N. vectensis* has both up and downstream cellular and molecular mechanisms associated with EMT described in bilaterians. However, *N. vectensis* does not segregate a distinct mesodermal germ layer nor display EMT under natural conditions. In bilaterians, this mechanism must have evolved to segregate mesodermal cells from the endoderm to retain the tight cell-cell junctions required in endodermal epithelia. Interestingly, mesoderm segregation via EMT in *Drosophila* takes place after epithelial folding in response to *snail* expression. In these embryos, contractile myosin enhances the localization of AJs and Par-3 in the presumptive mesoderm and prevents their downregulation by Snail, thus delaying EMT (Weng and Wieschaus, 2016, 2017). Furthermore, the overexpression of Snail in *Drosophila* embryos is sufficient to disassemble ectodermal-AJs, but mesodermal-AJs are maintained by actomyosin contraction that antagonize Snail effects (Weng and Wieschaus, 2016, 2017). Our results suggest a similar mechanism since *Nvsnail* overexpression in endomesodermal lineages (Figure 6—figure supplement 1) is not sufficient to segregate cells and the endomesoderm remains as an epithelium. However, unlike *Drosophila*, Par proteins and AJs are not enhanced but degraded during the gastrulation of *N. vectensis* (Figure 1). As it is discussed in (Weng and Wieschaus, 2017), not only the degradation but also the turnover of AJs and Par proteins in adjacent epithelia is essential for EMT-mediated germ layer segregation in different animals. The dual identity of *N. vectensis* endomesoderm is characterized by the continuous expression of *Nvsnail* genes (Martindale et al., 2004) that repress the turnover of AJs and may play a role in inhibiting EMT from occurring (Figures 6 and 7).

Interestingly, components of the Wnt/PCP pathway are expressed only in the endomesoderm (Kumburegama et al., 2011; Wijesena et al., 2011), while components of the Par system are expressed only in the ectoderm (Salinas-Saavedra et al., 2015). It could be that NvSnail degrades AJs and inhibits their re-assembly in the endomesoderm, but the activation of contractile myosin by the Wnt/PCP pathway maintains the endomesodermal cells together in *N. vectensis* embryos. Hence in bilaterians, a mechanism (most likely downstream of Snail) that connects the cytoskeleton with cell-polarity may have evolved to tighten cell-cell adhesion in the endoderm and allow EMT.

To elucidate this, further comparative research and funding are needed to understand the cellular mechanisms that evolve to segregate mesoderm and control epithelial cell polarity at the base of the metazoan tree. For example, cnidarians, poriferans, and ctenophores present intriguing characteristics to study. In cnidarians, different modes of gastrulation have been described between species including unipolar and multipolar cell ingression and delamination (Kirillova et al., 2018; Byrum and Martindale, 2004; Marlow and Martindale, 2007). Poriferans display EMT-like processes and cell morphologies during regeneration and trans-differentiation (Nakanishi et al., 2014; Coutinho et al., 2017). However, whether or not these processes involve similar molecular and cellular mechanisms are still unclear. Interestingly, ctenophores segregate a mesodermal cell population during embryogenesis but do not have the genes that encode for all cell-cell adhesion complexes and specify for mesoderm in bilaterians (Figure 8—figure supplement 1) (Ganot et al., 2015; Ryan et al., 2013). Thus, there is much to be learned by the comparative study of cell biology to understand the evolutionary origins of EMT and germ layer formation.

## Materials and methods

**Key resources table:** 

Reagent type (species) or resource	Designation	Source or reference	Identifiers	Additional information
Antibody	Mouse Anti-alpha-Tubulin Monoclonal Antibody, Unconjugated, Clone DM1A	Sigma-Aldrich	T9026; RRID:AB_477593	(1:500)
Antibody	Anti-beta-Catenin antibody produced in rabbit	Sigma-Aldrich	C2206; RRID:AB_476831	(1:300)
Antibody	Anti-Histone, H1 + core proteins, clone F152.C25.WJJ antibody	Millipore	MABE71; RRID:AB_10845941	(1:300)
Antibody	anti-NvaPKC custom peptide antibody produced in rabbit	Bethyl labs; Salinas-Saavedra et al. (2015)		Stored at MQ Martindale's lab; (1:100)
Antibody	anti-NvLgl custom peptide antibody produced in rabbit	Bethyl labs; Salinas-Saavedra et al. (2015)		Stored at MQ Martindale's lab; (1:100)
Antibody	anti-NvPar-1 custom peptide antibody produced in rabbit	Bethyl labs; Salinas-Saavedra et al. (2015)		Stored at MQ Martindale's lab; (1:100)
Antibody	anti-NvPar-6 custom peptide antibody produced in rabbit	Bethyl labs; Salinas-Saavedra et al. (2015)		Stored at MQ Martindale's lab; (1:100)
Antibody	Goat anti-Mouse IgG Secondary Antibody, Alexa Fluor 568	Thermo Fisher Scientific	A-11004; RRID:AB_2534072	(1:250)
Antibody	Goat anti-Rabbit IgG Secondary Antibody, Alexa Fluor 647	Thermo Fisher Scientific	A-21245; RRID:AB_2535813	(1:250)
Antibody	Sheep Anti-Digoxigenin Fab fragments Antibody, AP Conjugated, Roche	Roche	11093274910; RRID:AB_514497	
Other	Alexa Fluor 488 Phalloidin	Thermo Fisher Scientific	A12379; RRID:AB_2315147	(1:200)
Other	Texas Red Streptavidin	Vector Laboratories	SA-5006, RRID:AB_2336754	(1:200)
Other	DAPI (4',6-Diamidino- 2-Phenylindole, Dihydrochloride)	Thermo Fisher Scientific	D1306; RRID:AB_2629482	(0.1 µg/µl)
Chemical compound, drug	Protein kinase Cζ pseudosubstrate, myristoyl trifluoroacetate salt	Sigma-Aldrich	P1614	
Chemical compound, drug	1-Azakenpaullone	Sigma-Aldrich	A3734	
Chemical compound, drug	Biotinylated Dextran Amine-Texas Red	Vector Laboratories	SP-1140; RRID:AB_2336249	
Chemical compound, drug	Dextran, Alexa Fluor 488; 10,000 MW, Anionic, Fixable	Thermo Fisher Scientific	D22910	
Chemical compound, drug	Dextran, Alexa Fluor 555; 10,000 MW, Anionic, Fixable	Thermo Fisher Scientific	D34679	
Chemical compound, drug	Dextran, Alexa Fluor 647; 10,000 MW, Anionic, Fixable	Thermo Fisher Scientific	D22914	
Chemical compound, drug	Dextran, Cascade Blue, 10,000 MW, Anionic, Lysine Fixable	Thermo Fisher Scientific	D1976	
Deposited Data	*Nematostella vectensis* genome assembly 1.0	JGI	https://genome.jgi.doe.gov/Nemve1/Nemve1.home.html	
Biological sample	*Nematostella vectensis*	Whitney Laboratory for Marine Bioscience, FL, USA.	RRID:SCR_005153	
Sequence-based reagent	Genomic PCR *Nvpar-6* and dn*Nvpar-6*: F-AAAACCAC CATCAGCCGAGTCA; R-TATTGATAGAATACCAGTCTCA		NEMVEDRAFT _v1g233358	
Sequence-based reagent	sgRNAs *Nvpar-6*: 1-GGATGTTGCCGACTCGCAGT; 2-GGAGAAGGCGAACTCGTCTG; 3-GGATAACCCTGTGCCAGTCA	CRISPRevolution sgRNA; Synthego	NEMVEDRAFT _v1g233358	
Sequence-based reagent	dn*Nvpar-3*: F-ATGATGAAGGTTGTAGT; R-TGCGCCCGATTCGAATCCATCT		NEMVEDRAFT _v1g240248	
Sequence-based reagent	sgRNAs *Nvpar-3*: 1-GGGTGTTCGAGGGACGCGAT; 2- GGGCAGGTTTATCCCGAAGG; 3- ACCAACGAUCUAGAUCCAGU	CRISPRevolution sgRNA; Synthego	NEMVEDRAFT _v1g240248	
Sequence-based reagent	Genomic PCR *Nvpar-3*: F-GTAGACGGGACTGGTTTGGA; R-AGGGACAGGTTGCTCCTTTT		NEMVEDRAFT _v1g240248	
Sequence-based reagent	dn*Nvpar-1*: F-AATATAAACTATGAACTTAACG; R-TTAAAGTTTTAATTCATTTGCA		NEMVEDRAFT _v1g 139527	
Sequence-based reagent	sgRNAs *Nvsnail-A*: 2-GGGGCCGGTAATGACGCGCG; 3-GGCGTAGAGTCACACCGCAA; 4-GGCGATGATATCGAGCTCGG; 5-GGGCATCTTGAGTGCACCCA	CRISPRevolution sgRNA; Synthego	NEMVEDRAFT _v1g240686	
Sequence-based reagent	sgRNAs *Nvsnail-A*: 1-GGGCTCTCTTGCTCCGTAAC; 6-GGGTTTCCTGGCGCTGGGAT	CRISPRevolution Modified sgRNA; Synthego	NEMVEDRAFT _v1g240686	
Sequence-based reagent	Genomic PCR *Nvsnail-A* (full length): F-ATGCCCCGCTCGTTTCTAG; R-TCCTTGTGACGGGCAGCC		NEMVEDRAFT _v1g240686	
Sequence-based reagent	sgRNAs *Nvsnail-B*: 1-GGAAGAGGATGTGAGGTTTT; 2-GAGATGATATTAGGCTGGTG; 4-GAAAAGCTGTACGACTCCTT; 5-GGGCATCTTGAGAGCGCCCA	CRISPRevolution sgRNA; Synthego	NEMVEDRAFT _v1g236363	
Sequence-based reagent	sgRNAs *Nvsnail-B*: 3-GGGTGAAGACTAAGACAGAG; 6-GGGCGATGAATCGTGTTTAA	CRISPRevolution Modified sgRNA; Synthego	NEMVEDRAFT _v1g236363	
Sequence-based reagent	Genomic PCR *Nvsnail-B* (full length): F-ATGCCGAGGTCCTTCCTGG; R-GCAGAGATTTTGCCGACACAT		NEMVEDRAFT _v1g236363	
Recombinant DNA reagent	pSPE3-mVenus	Roure et al. (2007)		Gateway vector
Recombinant DNA reagent	pSPE3-mCherry	Roure et al. (2007)		Gateway vector
Recombinant DNA reagent	pSPE3*-Nvpar-6*-mVenus	Salinas-Saavedra et al. (2015)		
Recombinant DNA reagent	pSPE3-*Nvpar-3*-mVenus	Salinas-Saavedra et al. (2015)		
Recombinant DNA reagent	*Nv*ß-catenin expression constructs	Wikramanayake et al. (2003); Röttinger et al., 2012		
Recombinant DNA reagent	*Nvsnail-A* backbone to generate expression constructs	Magie et al. (2007)		
Software, algorithm	Fiji (ImageJ)	NIH	http://fiji.sc	
Software, algorithm	Imaris 7.6.4	Bitplane Inc.		
Software, algorithm	CRISPRscan	Moreno-Mateos et al. (2015)	http://www.crisprscan.org/	
Software, algorithm	SPSS	IBM		

### Culture and spawning of *Nematostella vectensis*

Spawning, gamete preparation, fertilization and embryo culturing of *N. vectensis* (RRID:SCR_005153) embryos was performed as previously described (Röttinger et al., 2012; Hand and Uhlinger, 1992; Layden et al., 2013; Wolenski et al., 2013). Adult *N. vectensis* were cultivated at the Whitney Laboratory for Marine Bioscience of the University of Florida (USA). Males and females were kept in separate glass bowls (250 ml) in 1/3x seawater (salinity: 12pp) reared in dark at 16°C. Animals were fed freshly hatched *Artemia* three times a week and macerated oyster the day before spawning. Spawning was induced by incubating the adults under an eight-hour light cycle at 25°C the night before the experiment. Distinct groups of animals were spawned once every 2 weeks. Oocytes and sperm were collected separately and fertilized in vitro by adding sperm to egg masses for 25 min. The jelly mass surrounding the fertilized eggs was removed by incubating the eggs in 4% L-Cysteine (in 1/3x seawater; pH 7.4) for 15–17 min and then washed 3 times with 1/3x seawater. De-jellied eggs were kept in glass dishes (to prevent sticking) in filtered 1/3 seawater at 16°C until the desired stage.

### Immunohistochemistry

All immunohistochemistry experiments were carried out using the previous protocol for *N. vectensis* (Salinas-Saavedra et al., 2015) with a slight modification in the glutaraldehyde concentration to allow better antibody penetration. Embryos were fixed on a rocking platform at room temperature in two consecutive steps. Embryos of different stages were fixed for no longer than 3 min in fresh Fix-1 (100 mM HEPES pH 6.9; 0.05M EGTA; 5 mM MgSO4; 200 mM NaCl; 1x PBS; 3.7% Formaldehyde; 0.2% Glutaraldehyde; 0.5% Triton X-100; and pure water). Then, Fix-1 was removed and replace with fresh Fix-2 (100 mM HEPES pH 6.9; 0.05M EGTA; 5 mM MgSO4; 200 mM NaCl; 1x PBS; 3.7% Formaldehyde; 0.05% Glutaraldehyde; 0.5% Triton X-100; and pure water). Embryos were incubated in Fix-2 for 1 hr. Fixed embryos were rinsed at least five times in PBT (PBS buffer plus 0.1% BSA and 0.2% Triton X-100) for a total period of 3 hr. PBT was replaced with 5% normal goat serum (NGS; diluted in PBT) and fixed embryos were blocked for 1 to 2 hr at room temperature with gentle rocking. Primary antibodies were diluted in 5% NGS to desired concentration. Blocking solution was removed and replaced with primary antibodies diluted in NGS. All antibodies incubations were conducted over night on a rocker at 4°C. After incubation of the primary antibodies, samples were washed at least five times with PBT for a total period of 3 hr. Secondary antibodies were then applied (1:250 in 5% NGS) and samples were left on a rocker overnight at 4°C. Samples were then washed with PBT and left on a rocker at room temperature for an hour. To visualize F-actin, samples were incubated then for 1.5 hr in Phalloidin (Invitrogen, Inc. Cat. # A12379) diluted 1:200 in PBT. Samples were then washed once with PBT and incubated with DAPI (0.1 µg/µl in PBT; Invitrogen, Inc. Cat. # D1306) for 1 hr to allow nuclear visualization. Stained samples were rinsed again in PBS two times and dehydrated quickly into isopropanol using the gradient 50, 75, 90, and 100%, and then mounted in Murray’s mounting media (MMM; 1:2 benzyl benzoate:benzyl alcohol) for visualization. Note that MMM may wash DAPI out of your sample. For single blastomere microinjection experiments, after Phalloidin staining, samples were incubated with Texas Red Streptavidin (1:200 in PBT from 1 mg/ml stock solution; Vector labs, Inc. Cat.# SA-5006. RRID:AB_2336754) for 1 hr to visualize the injected dextran. We scored more than 1000 embryos per each antibody staining and confocal imaged more than 50 embryos at each stage.

The primary antibodies and concentrations used were: mouse anti-alpha tubulin (1:500; Sigma-Aldrich, Inc. Cat.# T9026. RRID:AB_477593), rabbit anti-ß-catenin (1:300; Sigma-Aldrich, Inc. Cat.# C2206. RRID:AB_476831), mouse anti-histone H1 (1:300; F152.C25.WJJ, Millipore, Inc. RRID:AB_10845941).

Rabbit anti-*Nv*aPKC, rabbit anti-*Nv*Lgl, rabbit anti-*Nv*Par-1, and rabbit anti-*Nv*Par-6 antibodies are custom made high affinity-purified peptide antibodies that were previously raised by the same company (Bethyl Inc.). All these four antibodies are specific to *N. vectensis* proteins (Salinas-Saavedra et al., 2015) and were diluted 1:100.

Secondary antibodies are listed in Key resources table.

### Fluorescent tracer dye penetration assay

Primary polyps were incubated and mounted in 1/3 sea water with fluorescent dextran solution (0.5 mg/ml). For uninjected embryos we used Dextran, Alexa Fluor 555 (Molecular Probes, INC. Cat.# D34679). For injected embryos, expressing fluorescent proteins, we used Dextran, Alexa Fluor 647 (Molecular Probes, INC. Cat.# D22914). Animals were observed within 10 min of incubation. 15 animals were recorded per treatment. For better visualization of the dextran solution inside the gastric cavity as shown in Figure 2B, we delivered additional dextran solution by microinjecting dye through the polyp’s mouth. For the rest of the experiments, we mainly focused in the ectodermal permeability and we let the polyps to eat the solution by themselves as grown babies.

### mRNA microinjections

The coding region for each gene of interest was PCR-amplified and cloned into pSPE3-mVenus or pSPE3-mCherry using the Gateway system (Roure et al., 2007). Eggs were injected directly after fertilization as previously described (Salinas-Saavedra et al., 2015; Layden et al., 2013; DuBuc et al., 2014) with the mRNA encoding one or more proteins fused in frame with reporter fluorescent protein (N-terminal tag) using final concentrations of 450 ng/µl for each gene. Fluorescent dextran was also co-injected to visualize the embryos. For single blastomere microinjections, we raised the embryos until 8–16 cell stages (3–4 hpf) and co-injected the mRNA solution with Biotinylated Dextran Amine-Texas Red (10 µg/µl; Vector labs, Inc. Cat.# SP-1140. RRID:AB_2336249). Live embryos were kept at 16°C and visualized after the mRNA of the FP was translated into protein (2–3 hr). To avoid lethality, lower mRNA concentrations of the mutant proteins (250 ng/µl) were used to image the specimens for Figures 2 and 4, and Figure 3—video 1. Live embryos were mounted in 1/3 sea water for visualization. Images were documented at different stages from 3 to 96 hr post fertilization. We injected and recorded more than 500 embryos for each injected protein and confocal imaged approximately 20 specimens for each stage for detailed analysis of phenotypes *in vivo*. We repeated each experiment at least five times obtaining similar results for each case. The fluorescent dextran and primers for the cloned genes are listed in Key resources table.

### CRISPR/Cas9 knock-outs

To target our gene of interest, we used synthetic guide RNAs (sgRNA; Synthego, Inc.) and followed the instructions obtained from the manufacturer to form the RNP complex with Cas9 (Cas9 plus sgRNAs). Target sites, off-target sites, and CFD scores were identified and sgRNA were designed using CRISPRscan (Doench et al., 2014; Moreno-Mateos et al., 2015). We delivered the RNP complex by microinjection as previously described (Servetnick et al., 2017; Wijesena et al., 2017; Ikmi et al., 2014. Lyophilized Cas9 (PNA Bio., Inc. Cat.# CP01) was reconstituted in nuclease-free water with 20% glycerol to a final concentration of 2 µg/µl. Reconstituted Cas9 was aliquoted for single use and stored at −80°C. Embryos were injected, as described for mRNA microinjections, with a mixture (12.5 µl) containing sgRNAs (80 ng/μl of each sgRNA), Cas9 (3 μg), and Alexa Fluor 488-dextran (0.2 μg/μl; Molecular Probes, Inc. Cat.# D22910). Cas9 and sgRNA guides only controls were injected alongside each round of experiments. sgRNA guides controls are only shown in figures as Cas9 had no significative effects. 3 sgRNA were used to knock out *Nvpar-3*, 3 sgRNA were used to knock out *Nvpar-6*, 6 sgRNA were used to knock out *Nvsnail-A*, and 6 sgRNA were used to knock out *Nvsnail-B*. Single-embryo genomic DNA was analyzed as previously described (Servetnick et al., 2017). Gene expression was confirmed by in situ hybridization. We injected and recorded more than 1000 embryos for each treatment. We repeated each experiment at least six times obtaining similar results for each case. sgRNAs’ sequences and PCR primers flanking the targeted region are listed in Key resources table.

### In situ hybridization

In situ hybridization was carried out following a previously published protocol for *N. vectensis* (Wolenski et al., 2013). Animals were fixed in ice-cold 4% paraformaldehyde with 0.2% glutaraldehyde in 1/3x seawater for 2 min, followed by 4% paraformaldehyde in PBTw for 1 hr at 4°C. Digoxigenin (DIG)-labeled probes, previously described (Salinas-Saavedra et al., 2015; Röttinger et al., 2012), were hybridized at 63°C for 2 days and developed with the enzymatic reaction of NBT/BCIP as substrate for the alkaline phosphatase conjugated anti-DIG antibody (Roche, Inc. Cat.#11093274910. RRID:AB_514497). Samples were developed until gene expression was visible as a purple precipitate.

### Drug treatment

We incubated *N. vectensis* embryos in 20 µM of aPKC pseudosubstrate inhibitor (Protein kinase Cζ pseudosubstrate, myristoyl trifluoroacetate salt, Sigma, Cat.#P1614) from 0 to 4 hpf. Controls and 1-azakenpaullone (AZ; Sigma, Cat.#A3734) drug treatment of *N. vectensis* embryos was performed as previously described (Röttinger et al., 2012; Leclère et al., 2016). Embryos were developed in 5 µm AZ from 3 to 76 hpf. Controls were incubated in 0.08% DMSO.

### Imaging of *N. vectensis* embryos

Images of live and fixed embryos were taken using a confocal Zeiss LSM 710 microscope using a Zeiss C-Apochromat 40x water immersion objective (N.A. 1.20). Pinhole settings varied between 1.2 and 1.4 A.U. according to the experiment. The same settings were used for each individual experiment to compare control and experimental conditions. Results from in situ hybridization studies were imaged using a Zeiss Imager.M2 with a Zeiss 425 HRc color digital camera run by Zeiss Zen 2012 software. Z-stack images were processed using Imaris 7.6.4 (Bitplane Inc.) software for three-dimensional reconstructions and FIJI for single slice and movies. Final figures were assembled using Adobe Illustrator and Adobe Photoshop.

### Co-Immunoprecipitation

Tissue homogenization and protein extraction was performed as described in (Salinas-Saavedra et al., 2015; Suzuki et al., 2001; Wang et al., 2012). Briefly, embryos were homogenized in 200 µl of ice cold lysis buffer (30 mM HEPES, pH to 7.5, 1 mM EDTA, 150 mM NaCl, 50 mM NaF, 1 mM Na3VO4, 1 mM Na2MoO4, 1 mM MgCl2, 1% NP-40, 10% Glycerol, Protease Inhibitor cocktail (Sigma P8340) and PMSF. After 15 min’ incubation on ice, crude lysate was carefully laid on top of a 200 µl sucrose cushion (1M sucrose, 30 mM HEPES, pH to 7.5, 1 mM EDTA, 150 mM NaCl, 50 mM NaF, 1 mM Na3VO4, 1 mM Na2MoO4) and yolk pelleted by centrifugation at 1000 rpm for 10 min. The top layer was transferred to a clean microcentrifuge tube and 300 µl of lysis buffer was added. Approximately, 5 mg of protein was obtained from 60 µl of embryos (more than 15,000 embryos) homogenized in 500 µl of lysis buffer. 2 mg of total protein (in 500 µl of lysate buffer) was incubated with Par-specific antibodies cross-linked to Pierce Protein A/G Magnetic Beads (Pierce Biotechnology, Rockford, IL). (Pre-immune) IgG-IP pull downs were performed as a negative control for each experiment. Three antibodies, described previously (Salinas-Saavedra et al., 2015), against three different proteins (NvaPKC, NvPar-6, and NvPar-1) were utilized. We performed co-IP experiments using early cleavage (2–4 hpf) and for gastrula stages (24–30 hpf) lysates. Co-IP experiments were repeated four times for each stage using fresh lysates every time. For a detailed protocol, please, go to http://www.whitney.ufl.edu/research/faculty/mark-q-martindale/mark-q-martindale-lab-protocols/

### Morphometric measurements

Epithelial thickness was measured using confocal images of embryos immunohistochemically labeled, processed with Imaris 7.6.4 (Bitplane Inc.). For detailed and graphical explanation, please see Figure 3—figure supplement 3 and Figure 3—figure supplement 7. For each treatment, epithelial thickness was determined by the average cell length (µm) along the apico-basal axis (A-B axis) of five individual cells. These values were made for two perpendicular axes Axis1 (A1) and Axis2 (A2) and normalized by the embryonic diameter (µm), in order to minimize technical artifacts (e.g. fixation and mounting) that could have affected the shape/size of the cell. Giving a proportion (p) that was calculated for A1 and A2, named p1 and p2, respectively. The values of p1 and p2 obtained for 90 control embryos, were statistically compared with the respective values obtained for 103 mutant-embryos using the Mann–Whitney U test (nonparametric; normality was tested using SPSS software) with a critical p-value of 0.05. The null hypothesis assumed no differences between the cell sizes of control and mutant-embryos. Values and statistics can be found in Figure 3—figure supplement 3—source data 1.

## Additional files

Transparent reporting form

### Data availability

No major datasets were generated in this study. All data generated or analyzed during this study are included in the manuscript and supporting files. All raw images are available on request.

The following previously published dataset was used:

Putnam NHSrivastava MHellsten UDirks BChapman JSalamov ATerry AShapiro HLindquist EKapitonov VVJurka JGenikhovich GGrigoriev IVLucas SMSteele REFinnerty JRTechnau UMartindale MQRokhsar DS2007*Nematostella vectensis* genome assembly JGI 1.0http://genome.jgi.doe.gov/Nemve1/Nemve1.home.htmlPublicly available at the JGI Genome Portal
