# Supplementary figures and images for: Germ layer-specific regulation of cell polarity and adhesion gives insight into the evolution of mesoderm
Source: eLife. 2018 Jul 31;7:e36740. doi: 10.7554/eLife.36740 (PMC6067901; doi:10.7554/eLife.36740)

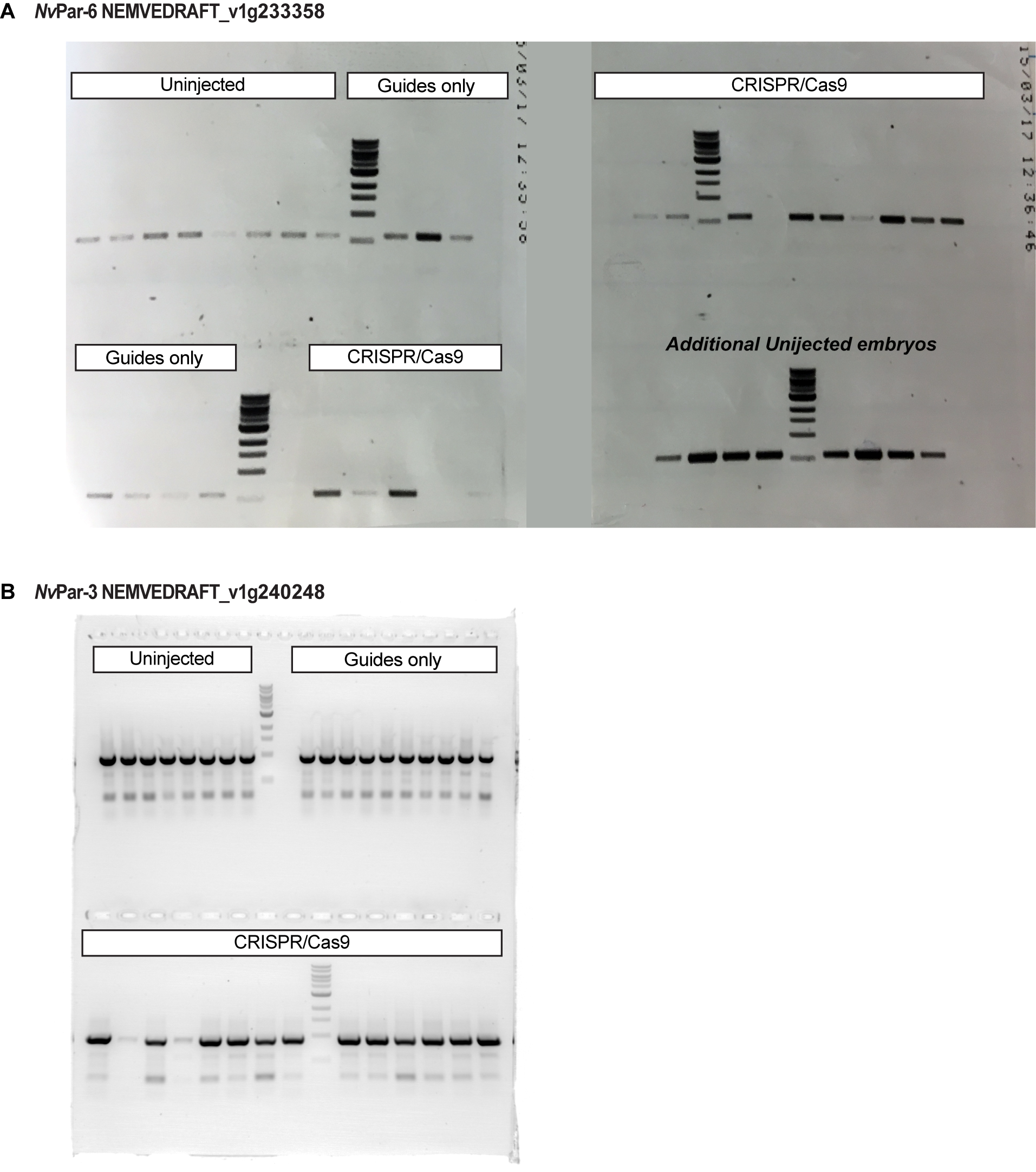

Supplement: Figure 3—figure supplement 4—source data 2. — White boxes correspond to the sections reported in Figure 3—figure supplement 4. [file elife-36740-fig3-figsupp4-data2.zip › Figure 3-figure supplement 4-Source Data 2.tif]

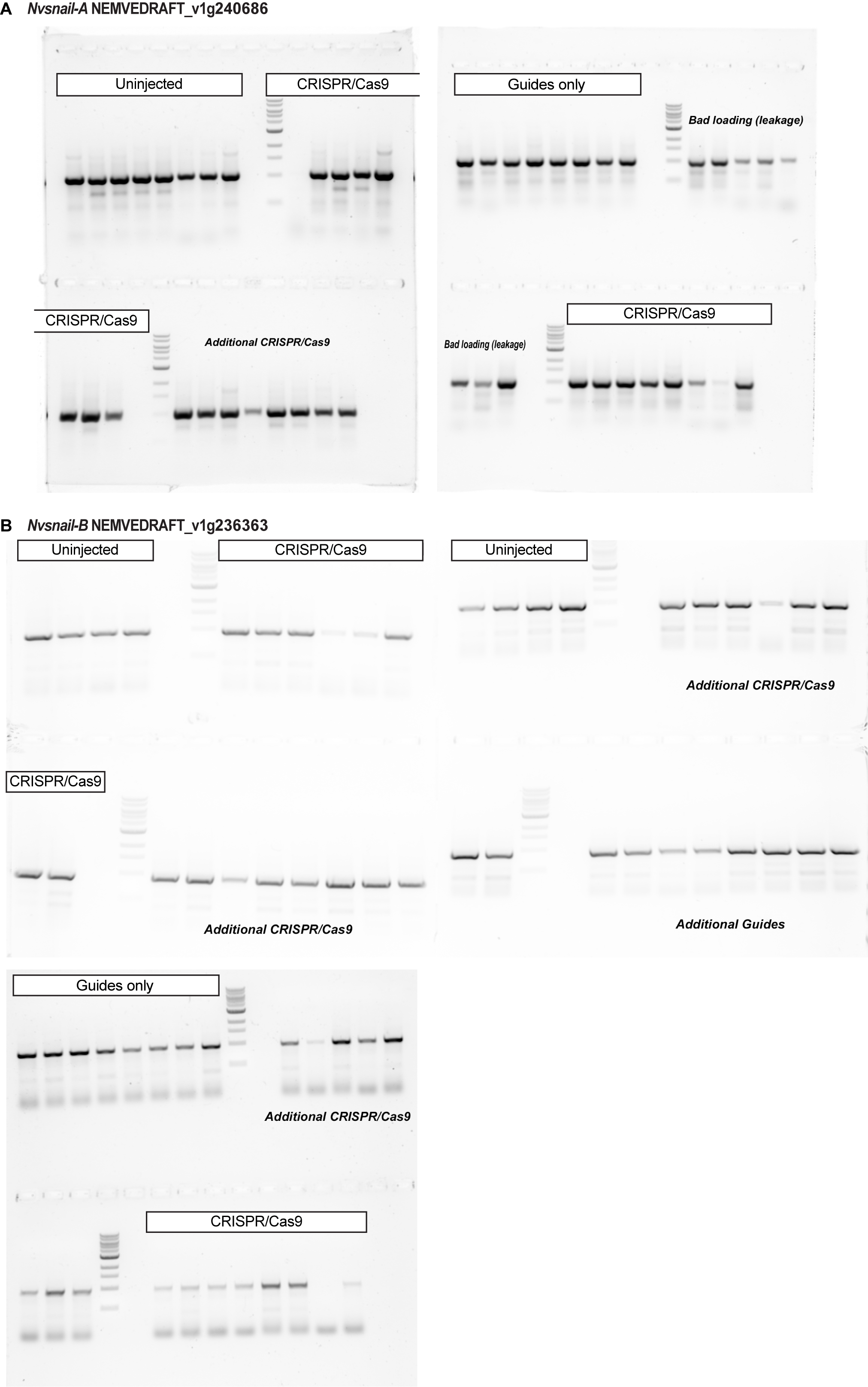

Supplement: Figure 7—figure supplement 2—source data 1. — White boxes correspond to the sections reported in Figure 7—figure supplement 2. [file elife-36740-fig7-figsupp2-data1.zip › Figure 7-figure supplement 2-Source Data 1.tif]
